# Circular RNA circZNF566 promotes hepatocellular carcinoma progression by sponging miR-4738-3p and regulating TDO2 expression

**DOI:** 10.1038/s41419-020-2616-8

**Published:** 2020-06-12

**Authors:** Shanbao Li, Junyong Weng, Fangbin Song, Lei Li, Chao Xiao, Weiqiang Yang, Junming Xu

**Affiliations:** 1 Department of General Surgery, Shanghai General Hospital, School of Medicine, Shanghai Jiaotong University, 200080 Shanghai, China; 2 Department of General Surgery, Jiading District Central Hospital Affiliated Shanghai University of Medicine & Health Sciences, Shanghai, China; 3 Department of Gastrointestinal Surgery, Changzheng Hospital, Second Military Medical University, 200003 Shanghai, China; 4 Shenzhen-Peking University-the Hong Kong University of Science and Technology Medical Center, Peking University Shenzhen Hospital, Shenzhen, China; 5 Department of General Surgery, Huashan Hospital, Fudan University, 200040 Shanghai, China

**Keywords:** Cancer therapy, Cell signalling

## Abstract

As a recently discovered noncoding RNA, circular RNAs (circRNAs) have been identified to play key roles in cancer biology; however, the detailed functions and mechanisms of circRNAs in hepatocellular carcinoma (HCC) remain largely unclarified. RNA-seq analysis was used to screen the expression profiles of circRNAs in HCC. CircZNF566 expression in HCC tissues and cell lines was detected by qRT-PCR. In vitro CCK-8, colony formation, wound healing, transwell migration, and invasion assays and in vivo tumorigenesis and metastasis assays were conducted to determine the functions of circZNF566. Luciferase reporter, RNA immunoprecipitation (RIP) and RNA pull-down assays were also performed to confirm the relationship between circZNF566 and miR-4738-3p. Bioinformatics analysis and luciferase reporter assays were employed to determine whether miR-4738-3p regulates tryptophan 2,3-dioxygenase (TDO2) expression. Finally, immunohistochemistry (IHC) was used to detect the level of TDO2 and determine its prognostic value. CircZNF566 was significantly upregulated in HCC tissues and cell lines. High circZNF566 expression in HCC tissues was positively correlated with clinicopathological features and poor prognosis. Functionally, in vitro experiments showed that circZNF566 promoted HCC cell migration, invasion, and proliferation, whereas in vivo experiments showed that circZNF566 promoted tumorigenesis and metastasis. Mechanistically, circZNF566 acted as a miR-4738-3p sponge to relieve the repressive effect of miR-4738-3p on its target TDO2. In addition, miR-4738-3p suppressed HCC cell migration, invasion, and proliferation, while TDO2 was positively correlated with pathological features and poor prognosis and promoted cell migration, invasion, and proliferation in HCC. CircZNF566 is a novel tumor promoter in HCC and functions through the circZNF566/ miR-4738-3p /TDO2 axis; in addition, circZNF566 may serve as a novel diagnostic marker, prognostic indicator, and target for the treatment of HCC.

## Introduction

According to cancer statistics, liver cancer is one of the most fatal cancers and the mortality of liver cancer has rapidly increased^1^. Hepatocellular carcinoma (HCC) accounted for 90% of liver cancers in China, and the incidence and mortality of HCC in China ranked fourth and third, respectively, among all cancers^2^. Although diagnostic tools and treatments have improved, HCC still has high rates of relapse, is prone to distant metastasis, and has a poor prognosis^3,4^. Some clinical biomarkers and new targets are being discovered to develop a more powerful therapeutic approach. However, the molecular pathogenesis of HCC is still complicated and poorly understood^5,6^. These challenges make it critical to urgently identify potential biomarkers for prognostic prediction and to find new targets for designing more effective treatments.

Circular RNAs (circRNAs) are a novel class of endogenous noncoding RNAs that are covalently closed loops of pre-mRNA transcripts with neither 5’ to 3’ polarity nor a polyadenylated tail. CircRNAs are ubiquitously expressed in many tumor tissues, such as liver, gastric, and breast cancer, and can regulate gene expression in mammals^7–10^. CircRNAs are usually stable, often conserved and comprise exons, introns, or both elements^11,12^. Natural endogenous circRNAs are inherently resistant to exonucleolytic RNA decay and contain selectively conserved microRNA (miRNA) target sites, so circRNAs can either act as “miRNA sponges” and competitively bind miRNAs to regulate posttranscriptional activity or interact with RNA polymerase II in the nucleus to regulate transcription^9,11,13^. These findings suggest that circRNAs might be a potential biomarker and therapeutic target for cancer.

Tryptophan 2,3-dioxygenase (TDO, EC 1.13.11.11) is a homotetrameric heme-containing cytosolic enzyme that is thought to be expressed mainly in liver and to a much reduced extent in the central nervous system and is encoded by the gene TDO2; TDO is the rate-limiting enzyme in the first step of tryptophan (Try) metabolism and can convert Try to produce kynurenine (Kyn)^14,15^. TDO has been implicated as a key regulator of neurotoxicity involved in neurodegenerative diseases, and could inhibit the growth of bacteria, parasites, and viruses when it was highly expressed^16–18^. Recently, it has been reported TDO is expressed in human tumors, such as human glioma cells, hepatocarcinomas, breast cancer, and some other tumors. In fact, of all cancers, TDO2 is most highly expressed in HCC^19–21^. TDO regulates tumor activity and the immune response via the Try-Kyn-aryl hydrocarbon receptor (Ahr) pathway, and similar research has also been reported in breast cancer^22,23^.

In our study, we analyzed the expression of circRNAs in HCC tissues and identified the novel circRNA circZNF566. CircZNF566 was not only upregulated in both HCC cells and tissues, but also closely related to the prognosis and clinicopathological characteristics of HCC patients, including clinical stage, distant metastasis. Importantly, circZNF566 significantly enhanced the progression and metastasis of HCC by sponging miR-4738-3p and targeting TDO2. Therefore, circZNF566 may serve as both a biomarker for predicting the prognosis and a potential therapeutic target for HCC patients.

## Results

### CircZN566 is upregulated in HCC cell lines and tissues and correlated with progression and poor prognosis

**Fig. 1:**
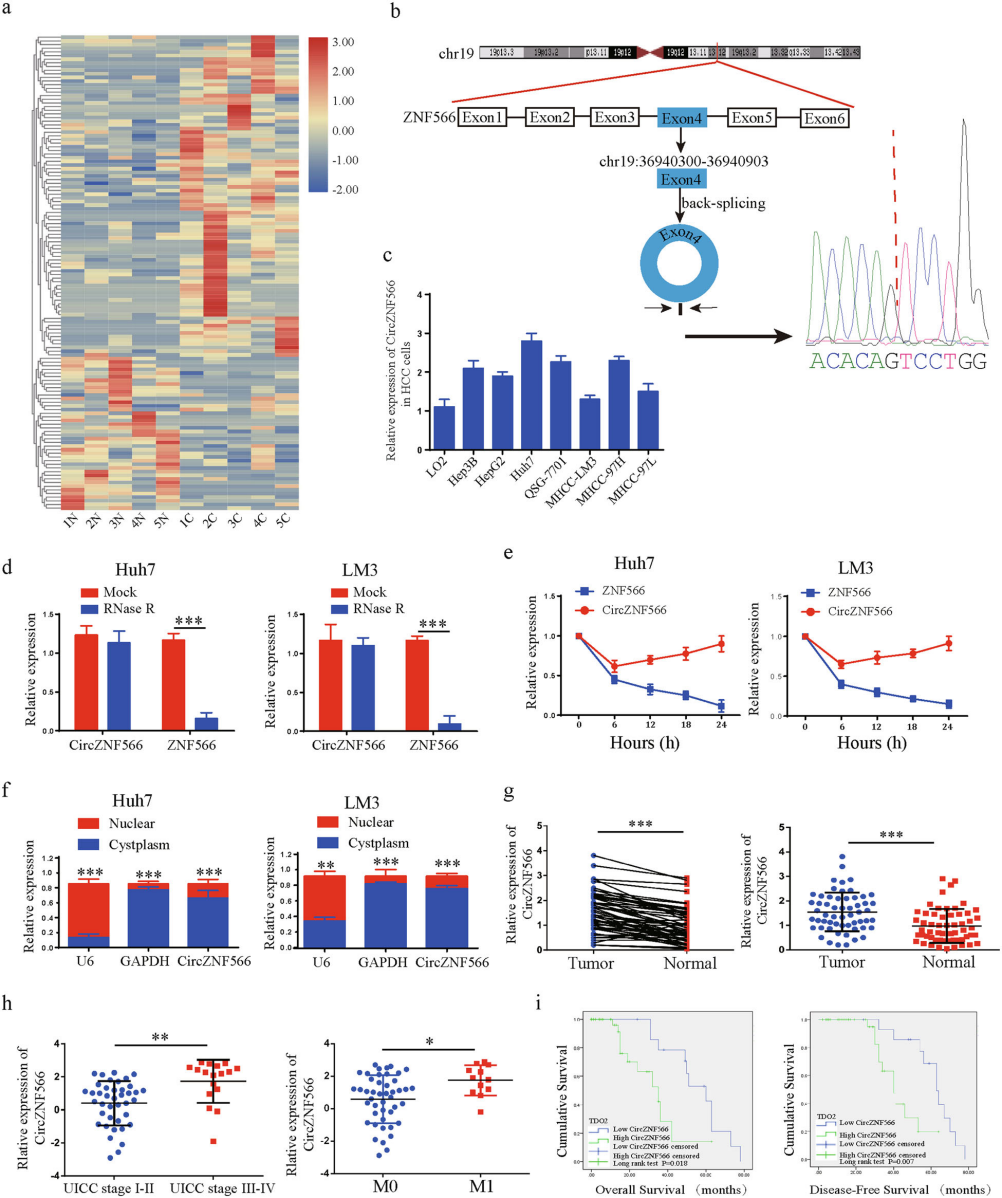
Validation and expression of circZNF566 in HCC tissues and cells. **a** CircRNA microarray comprising five pairs of HCC tissues and matched normal liver tissues. **b** Schematic illustration of the formation of circZNF566 via circularization of exons in the ZNF566 gene. The head-to-tail splicing of circZNF566 was confirmed by Sanger sequencing. **c** CircZNF566 expression in HCC and normal liver cell lines was detected by qRT-PCR. **d** The expression of circZNF566 and ZNF566 mRNA in both Huh7 and LM3 cells was validated by qRT-PCR in the presence or absence of RNase R. **e** qRT-PCR analysis of circZNF566 and ZNF566 mRNA after treatment with actinomycin D at the indicated time points in Huh7 and LM3 cells. **f** CircZNF566 was predominantly localized to the cytoplasm as indicated by a nuclear-cytoplasmic fractionation assay. **g** Analysis of the relatively differential expression levels of circZNF566 between 57 pairs of fresh frozen HCC and matched normal tissues. **h** The level of circZNF566 was obviously higher in stage M1 HCC tissues than in stage M0 HCC tissues and in UICC stage III–IV tumors than in UICC stage I–II tumors. **i** Kaplan–Meier survival analysis showed that HCC patients with high circZNF566 expression had a lower OS (*p* = 0.018) and DFS (*p* = 0.007) than those with low circZNF566 expression. All data are from three independent experiments and are presented as the means ± SEM or representative of three independent experiments with similar results (*p < 0.05, **p < 0.01, ***p < 0.001).

RNA-seq analysis of five matched pairs of HCC tissues and normal tissues was performed to characterize the expression of circRNA transcripts (Fig. 1a). According to the circBase annotation (http://www.circbase.org/), hsa-circ-0109500 (also named hsa-circ-0141434) was the most upregulated circRNA in HCC tissues; hsa-circ-0109500 is spliced from the ZNF566 gene and forms a sense-overlapping circular transcript of 603 nt in length (chr19:36940300-36940903), termed circZNF566, Sanger sequencing confirmed the head-to-tail splicing (Fig. 1b). CircZNF566 expression was obviously higher in the seven HCC cell lines (the Huh7 cells had the highest expression, the LM3 cells was the lowest) than in normal Lo2 cell line (Fig. 1c). To confirm the stability of circZNF566, we treated Huh7 and LM3 cells by RNase R and found that the linear ZNF566 levels decreased significantly, but circZNF566 resisted of RNase R digestion (Fig. 1d). The relative quantification of ZNF566 mRNA and circZNF566 results indicated that circZNF566 is about 40% of mRNA that undergoes to back splicing in both HCC cells (Supplementary Fig. S1a). In addition, after treatment with actinomycin D, the half-life of the circZNF566 transcript exceeded 24 h, while that of linear ZNF566 was ~6 h in both cell lines (Fig. 1e). Moreover, the results of nuclear-cytoplasmic fractionation revealed that circZNF566 was mainly localized in the cytoplasm (Fig. 1f). These results suggest that this macromolecule has the potential to be a diagnostic or prognostic marker for HCC. CircZNF566 expression was detected by qRT-PCR in 57 pairs of fresh frozen HCC tissues and matched normal liver tissues and we found that circZNF566 expression was higher in the HCC tissues (78.95%, 45/57) (Fig. 1g, Supplementary Fig. S1b). To further explore the relationship between circZNF566 and the clinicopathological features and prognosis of HCC, we divided the samples into a high and low circZNF566 group. As shown in (Table 1), high circZNF566 expression was associated with tumor size (*p* = 0.035), tumor differentiation (*p* = 0.011), and M stage (*p* = 0.016), but not with sex, age, tumor number, liver cirrhosis, or hepatitis virus (*p* > 0.05). CircZNF566 expression in UICC stage I–II (*p* = 0.0011) was obviously lower than that in UICC stage III–IV (*p* = 0.0138). Additionally, circZNF566 expression was higher in tissues with stage M1 than in those with stage M0 (Fig. 1h). HCC patients with high circZNF566 expression had significantly worse overall survival (OS) and disease-free survival (DFS) (Fig. 1i). Taken together, these findings reveal that circZNF566 is a stable circRNA and may be a potential biomarker for the clinical diagnosis and evaluation of HCC.

**Table 1:** Correlation between circZNF566 expression and clinicopathological features in HCC tissues (*n* = 57, *χ*^2^-test).

	N	CicrZNF566	Expression	*P*-value
		High (%)	Low (%)	
Age (year)				0.520
<60	45	24 (53.3%)	21 (46.7%)	
≥60	12	8 (66.7%)	4 (33.3%)	
Gender				0.092
Male	46	26 (71.7%)	20 (90.9%)	
Female	11	6 (55.5%)	5 (45.5%)	
Tumor number				0.502
Single	49	27 (55.1%)	22 (44.9%)	
Multiple	8	5 (62.5%)	3 (37.5%)	
Tumor size				0.035
<5 cm	27	16 (59.3%)	11 (40.7%)	
≥5 cm	30	21 (70.0%)	9 (30.0%)	
Tumor differentiation				0.011
I–II	37	16 (51.8%)	21 (43.2%)	
III–IV	20	16 (80.0%)	4 (20.0%)	
Tumor location				0.856
Left liver	10	5 (50.0%)	5 (50.0%)	
Right liver	42	25 (59.5%)	17 (40.5%)	
Whole liver	5	3 (60.0%)	2 (40.0%)	
Vascular invasion				0.085
Yes	16	12 (75.0%)	4 (25.0%)	
No	41	21 (51.2%)	20 (48.8%)	
Liver cirrhosis				1.000
Yes	29	17 (58.6%)	12 (41.4%)	
No	28	16 (57.1%)	12 (42.9%)	
Hepatitis virus				0.227
Yes	6	4 (66.7%)	2 (33.3%)	
No	51	31 (60.8%)	20 (39.2%)	
M stage				0.016
M0	46	24 (52.2%)	22 (47.8%)	
M1	11	10 (90.9%)	1 (9.1%)	

### CircZN566 promotes the mobility, migration, invasion, and proliferation of HCC cells

**Fig. 2:**
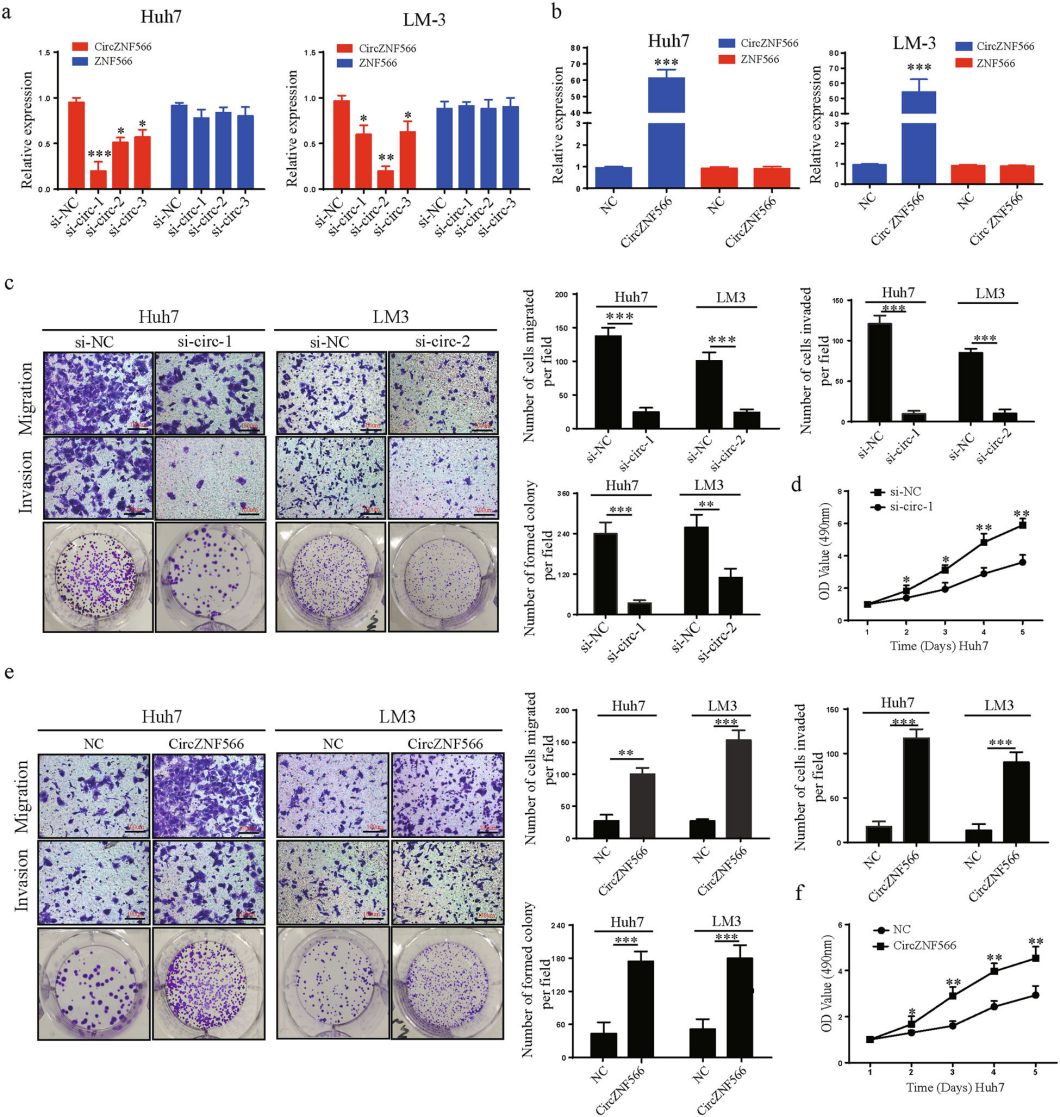
CircZNF566 promotes the mobility, migration, invasion, and proliferation of HCC cells in vitro. **a**, **b** The relative expression levels of circZNF566 and ZNF566 in HCC cells transfected with si-circZNF566-1, si-circZNF566-2, si-circZNF566-3, circZNF566 or NC were detected by qRT-PCR. **c** Migration and invasion of Huh7 and LM3 cells with knockdown of circZNF566 were evaluated by the transwell migration and invasion assays and the colony formation assay and the CCK-8 assay. **d** Proliferation of Huh7 cells with knockdown of circZNF566 were evaluated by CCK-8 assay. **e** Migration and invasion in Huh7 and LM3 cells that overexpressed circZNF566 were evaluated by the transwell migration and invasion assays and the colony formation assay. **e** Proliferation of Huh7 cells that overexpressed circZNF566 were evaluated by CCK-8 assay. All data are from three independent experiments and are presented as the means ± SEM or representative of three independent experiments with similar results (*p < 0.05, **p < 0.01, ***p < 0.001).

To explore the functions of circZNF566, we used three siRNAs targeting circZNF566 and overexpression plasmids, and then transfected them into Huh7 and LM3 cells. CircZNF566 expression was obviously silenced in both cells transfected with siRNA, while the ZNF566 mRNA level did not change (Fig. 2a). The si-circ-1 had the highest knockdown efficiency in Huh7 cells, in LM3 cells was si-circ-2 (Fig. 2a). By contrast, circZNF566 expression was obviously elevated in both HCC cell types transfected with the overexpression plasmids, but the ZNF566 mRNA did not change (Fig. 2b). When circZNF566 expression was downregulated, the mobility, migration, and invasion of HCC cells obviously decreased (Fig. 2c, Supplementary Fig. S2a). Moreover, silencing circZNF566 could significantly suppress the colony-forming ability and proliferation of Huh7 cells (Fig. 2c, d, Supplementary Fig. S2b). However, circZNF566 overexpression increased these results of LM3 cells (Fig. 2e, f, Supplementary Fig. S2c, d). In summary, these findings suggest that circZNF566 promotes the progression of HCC in vitro.

### CircZNF566 functions as an efficient miR-4738-3p sponge in HCC

**Fig. 3:**
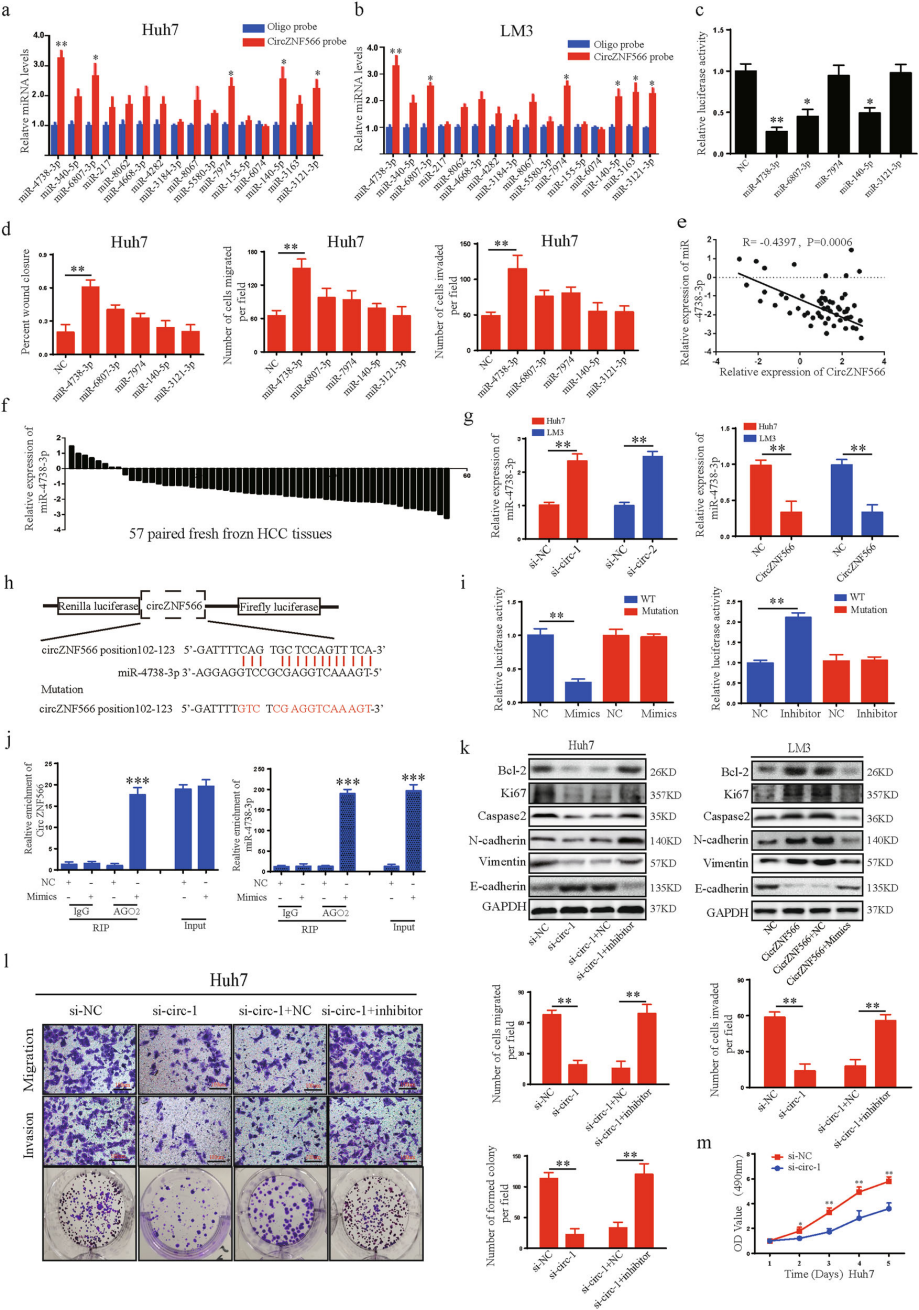
CircZNF566 promotes HCC progression by serving as an miRNA sponge of miR-4738-3p. **a**, **b** The relative abundances of 10 miRNA candidates in the Huh7 and LM3 lysates with circZNF566 or obligo probe were examined by qRT-PCR. **c** Luciferase activities of luc-circZNF566 in HCC cells transfected with five selected miRNA mimics or NC mimic were determined by a luciferase reporter assay. **d** Wound healing and transwell migration and invasion assays were performed to analyze the mobility, migration and invasion, respectively, of Huh7 cells transfected with five selected miRNA mimics or NC mimic. **e** The obviously negative correlation between the levels of circZNF566 and miR-4738-3p in 57 pairs of fresh frozen HCC tissues and matched normal liver tissues was analyzed by Pearson correlation analysis. **f** Relative miR-4738-3p expression in 57 pairs of fresh frozen HCC tissues and matched normal liver tissues. **g** The relative levels of miR-4738-3p in Huh7 and LM3 cell lines transfected with si-circZNF566, circZNF566 or NC were detected by qRT-PCR. **h** Schematic illustration of the sequence of wild-type TDO2 3’-UTR (WT) and TDO2 3’-UTR with mutations at the miR-4738-3p binding sites (mutant). **i** The effects of miR-4738-3p mimics, inhibitor and NC on luciferase activity were detected in HCC cells transfected with luciferase reporter plasmids expressing the WT 3’-UTR, mutant 3’-UTR or NC. **j** RIP assay for circZNF566 was performed with an anti-AGO2 antibody in HCC cells transfected with mimics or NC, and the expression of circZNF566 and miR-4738-3p was detected by qRT-PCR. **k** The effects of circZNF566 and miR-4738-3p on the protein expression of Bcl-2, Ki67, Caspase2, N-cadherin, E-cadherin, and Vimentin were detected by WB. **l** The effects of circZNF566 and miR-4738-3p on Huh7 cells migration, invasion and proliferation were evaluated by the transwell migration and invasion assays, the colony formation assay and the CCK-8 assay. All data are from three independent experiments and are presented as the means ± SEM or representative of three independent experiments with similar results (*p < 0.05, **p < 0.01, ***p < 0.001).

CircRNAs act as miRNA sponges and subsequently eliminate the function of their corresponding miRNAs. In this study, circZNF566 was mainly localized in the cytoplasm and exhibited high stability and suggested that circZNF566 may function as a miRNA sponge. Through bioinformatics analysis, we found that circZNF566 contains binding sites of multiple miRNAs, and then selected the top 10 scores for further study. A pull-down assay with a biotinylated circZNF566 probe was performed. Five possible miRNAs with significantly enhanced fold-changes for circZNF566 capture were observed in both HCC cells (Fig. 3a, b). To test the regulation of the above miRNAs on circZNF566, full-length circZNF566 was cloned into luciferase plasmids and transfected into cells with mimics of the above miRNAs. The luciferase activity was the lowest in the miR-4738-3p mimic group (Fig. 3c). Then detected the effects of these miRNAs on the mobility, migration, invasion, and proliferation of HCC cells and found that the miR-4738-3p group had increased these abilities (Fig. 3d, Sup: Fig. S3a). To detect miR-4738-3p expression in HCC tissues, we observed that miR-4738-3p was expressed at lower levels in HCC tissues (85.96%) (49/57) than in adjacent normal liver tissues (Fig. 3f). MiR-4738-3p expression in HCC tissues negatively correlated with tumor size (*p* = 0.022), tumor differentiation (*p* = 0.003), and M stage (*p* = 0.017) (Table 2). Next, the circZNF566 level negatively correlated with the level of miR-4738-3p in HCC tissues (Fig. 3e). Knockdown or overexpression of circZNF566 resulted in up- or downregulation of miR-4738-3p in HCC cells, respectively (Fig. 3g). Luciferase reporter plasmids containing the wild-type circZNF566 sequence (WT) or the circZNF566 sequence with mutations in the miR-4738-3p binding sites (mutant) were generated (Fig. 3h). Treatment with miR-4738-3p mimics inhibited the luciferase activity of the WT plasmid, while the miR-4738-3p inhibitor increased the luciferase activity of the WT plasmid; however, the luciferase activity of the mutant plasmid did not change with either treatment, which indicated a negative relationship between circZNF566 and miR-4738-3p (Fig. 3i). MiRNAs usually bind to microRNA response elements (MREs) in the RNA-induced silencing complex (RISC), of which the Argonaute2 (AGO2) protein is the key component. Thus, an anti-AGO2 RIP assay was performed. CircZNF566 and miR-4738-3p were immunoprecipitated from Huh7 cell lysates with an anti-AGO2 antibody. Compared with IgG, anti-AGO2 antibody could efficiently pull-down miR-4738-3p and circZNF566; furthermore, the amount of immunoprecipitated circZNF566 was significantly higher in the miR-4738-3p overexpression samples than in the NC samples (Fig. 3j). In conclusion, these results indicated that circZNF566 acts as a sponge of miR-4738-3p in HCC.

**Table 2:** Correlation between miR-4738-3p expression and clinicopathological features in HCC tissues (*n* = 57, *χ*^2^-test).

	N	miR-4738-3p	Expression	*P*-value
		High (%)	Low (%)	
Age (year)				0.258
<60	45	20 (44.4%)	25 (55.6%)	
≥60	12	7 (58.3%)	5 (41.7%)	
Gender				0.261
Male	46	22 (47.8%)	24 (52.2%)	
Female	11	5 (45.5%)	6 (54.5%)	
Tumor number				0.132
Single	49	21 (42.9%)	28 (57.1%)	
Multiple	8	2 (25.0%)	6 (75.0%)	
Tumor size				0.022
<5 cm	27	10 (37.0%)	17 (63.0%)	
≥5 cm	30	20 (66.7%)	10 (33.3%)	
Tumor differentiation				0.003
I–II	37	12 (32.4%)	25 (67.6%)	
III–IV	20	5 (25.0%)	15 (75.0%)	
Tumor location				>0.05
Left liver	10	7 (30.0%)	3 (70.0%)	
Right liver	42	20 (47.6%)	22 (52.4%)	
Whole liver	5	2 (40.0%)	3 (60.0%)	
Vascular invasion				>0.05
Yes	16	8 (50.0%)	8 (50.0%)	
No	41	19 (46.3%)	22 (53.7%)	
Liver cirrhosis				0.431
Yes	29	12 (58.6%)	17 (41.4%)	
No	28	13 (46.4%)	15 (53.6%)	
Hepatitis virus				0.408
Yes	6	2 (33.3%)	4 (66.7%)	
No	51	23 (45.1%)	28 (54.9%)	
M stage				0.017
M0	46	18 (39.1%)	28 (61.9%)	
M1	11	2 (18.2%)	9 (81.8%)	

### MiR-4738-3p reverses the ability of circZNF566 to promote HCC progression

To investigate whether circZNF566 exerts its biological function by sponging miR-4738-3p, rescue experiments were performed with upregulation or downregulation of miR-4738-3p in the presence of ectopic circZNF566 expression. MiR-4738-3p reversed the ability of circZNF566 to enhance the protein and mRNA expression of Bcl-2, Ki67, Caspase, N-cadherin, and Vimentin in HCC cells and reduce the protein and mRNA expression of E-cadherin (Fig. 3k, Supplementary Fig. S3f, g, h). Moreover, miR-4738-3p also reversed the ability of circZNF566 to promote the mobility, migration, invasion, and proliferation of HCC cells (Fig. 3l, m, Supplementary Fig. S3b–e). In conclusion, the ability of circZNF566 to promote HCC progression was reversed by miR-4738-3p.

### TDO2 is a direct target of miR-4738-3p

**Fig. 4:**
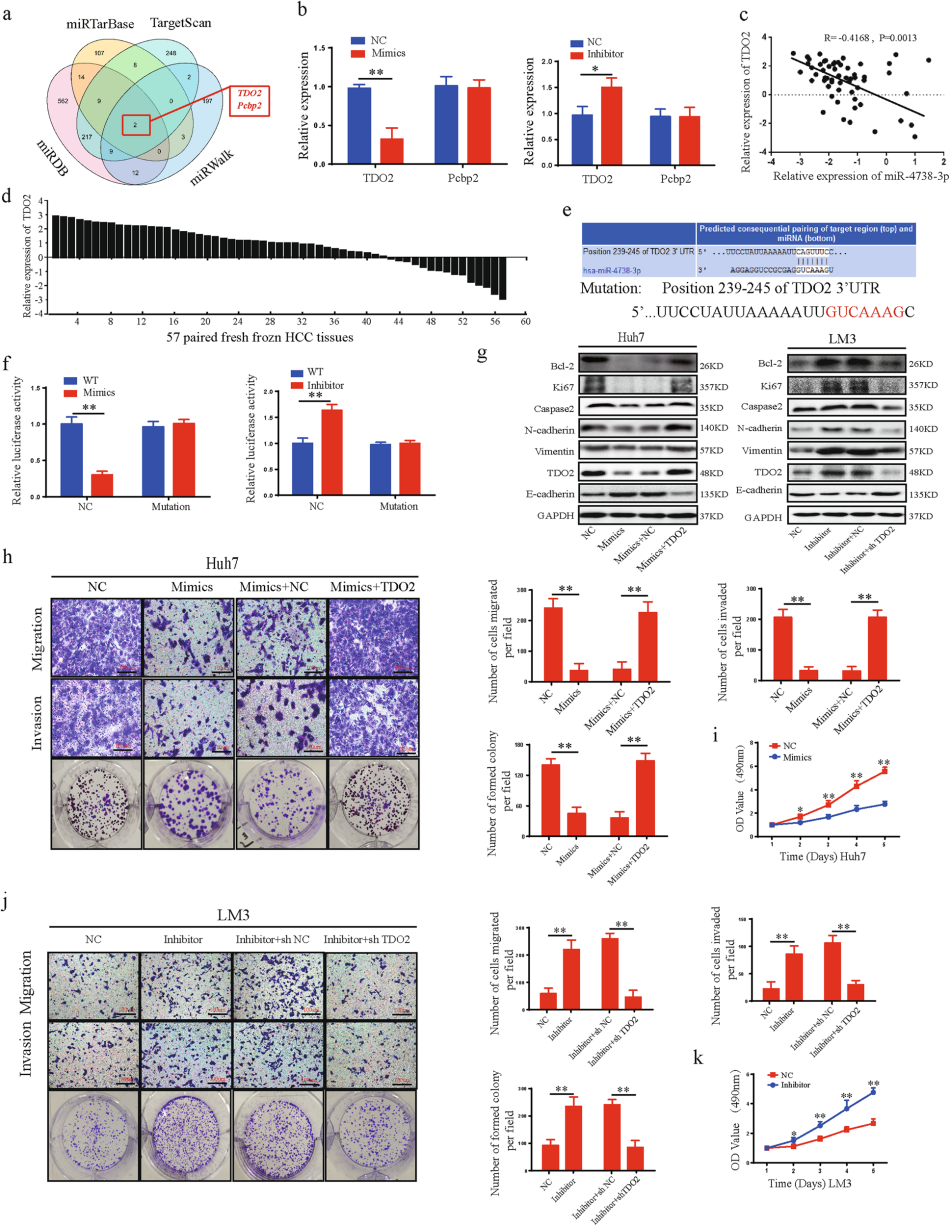
MiR-4738-3p suppresses HCC progression by directly targeting TDO2. **a** Schematic illustration exhibiting overlapping of the target mRNAs of miR-4738-3p predicted by miRTarBase, miRDB, TargetScan, and miWalk database. **b** TDO2 and Pcbp2 expression were detected in HCC cells treated with mimics, inhibitor or NC. **c** A negative relationship between the levels of miR-4738-3p and TDO2 was identified in 57 paired HCC tissues and normal liver tissues by Pearson correlation analysis. **d** Relative TDO2 expression in 57 pairs of fresh frozen HCC tissues and matched normal liver tissues. **e** The prediction of miR-4738-3p binding sites on the TDO2 mRNA 3′-UTR based on the TargetScan database. **f** The effects of miR-4738-3p mimics and inhibitor on the luciferase activities of wild-type TDO2 mRNA 3’-UTR (WT) and mutant TDO2 mRNA 3’-UTR (mutant) were detected. **g** The effects of miR-4738-3p and TDO2 on the protein expression of TDO2, Bcl-2, Ki67, Caspase2, N-cadherin, E-cadherin, and vimentin were detected by WB. **h–k** The effects of miR-4738-3p and TDO2 on Huh7 and LM3 cells migration, invasion, and proliferation were evaluated by transwell migration and invasion, colony formation and CCK-8 assays. All data are from three independent experiments and are presented as the means ± SEM or representative of three independent experiments with similar results (*p < 0.05, **p < 0.01, ***p < 0.001).

The miRTarBase, miRDB, TargetScan, and miWalk databases were used to predict the potential target genes of miR-4738-3p. Two genes, TDO2 and Pecbp2, were identified in all four databases as containing binding sites for miR-4738-3p (Fig. 4a). Overexpression of miR-4738-3p significantly decreased the mRNA and protein expression of TDO2 but not Pecbp2 in HCC cells, indicating that TDO2 may be the target gene of miR-4738-3p (Fig. 4b, Sup: Fig. S4a). The relationship of miR-4738-3p and TDO2 was analyzed and we found that TDO2 was upregulated in 77.19% (44/57) of HCC tissues and the miR-4738-3p level was negatively correlated with TDO2 in HCC tissues (Fig. 4c, d). Then, luciferase reporter plasmids with the 3’-UTRs of wild-type TDO2 mRNA (WT) and of TDO2 mRNA containing mutations in the miR-4738-3p binding sites (mutant) were constructed (Fig. 4e). The miR-4738-3p mimics significantly decreased the luciferase activity of the WT plasmid, and the miR-4738-3p inhibitor significantly increased the luciferase activity in cells expressing the WT plasmid but not in those expressing the mutant plasmid (Fig. 4f). These data suggested that miR-4738-3p suppresses TDO2 expression by directly binding to the 3’-UTR of TDO2 mRNA.

### MiR-4738-3p suppresses HCC progression by inhibiting TDO2 expression

Given the roles of circZNF566 in promoting HCC progression, we detected the role of miR-4738-3p on the mobility, migration, invasion, and proliferation of HCC cells. MiR-4738-3p mimics suppressed the mRNA and protein expression of TDO2, Bcl-2, Ki67, N-cadherin, Caspase, and Vimentin, and increase expression of E-cadherin while the miR-4738-3p inhibitor exerted opposing effects (Fig. 4g, Supplementary Fig. S4b). Functionally, miR-4738-3p mimics inhibited the mobility, migration, invasion, and proliferation of HCC cells (Fig. 4h, i, Supplementary Fig. S4d, e), while the miR-4738-3p inhibitor promoted these abilities (Fig. 4h, i, Supplementary Fig. S4d, e). To explore whether miR-4738-3p exerts its biological function by suppressing TDO2, we performed rescued experiments and found that TDO2 reversed the ability of miR-4738-3p to suppress the mRNA and protein expression of Bcl-2, Ki67, N-cadherin, Caspase, and Vimentin, and increase the expression of E-cadherin (Fig. 4g, Supplementary Fig. S4c). Functionally, TDO2 reversed the ability of miR-4738-3p to inhibit the mobility, migration, invasion, and proliferation of HCC cells (Fig. 4j, k, Supplementary Fig. S4f, g). In summary, these data suggest that miR-4738-3p suppresses HCC progression by inhibiting TDO2 expression.

### TDO2 is upregulated in HCC and positively correlated with clinicopathological characteristics and prognosis

**Fig. 5:**
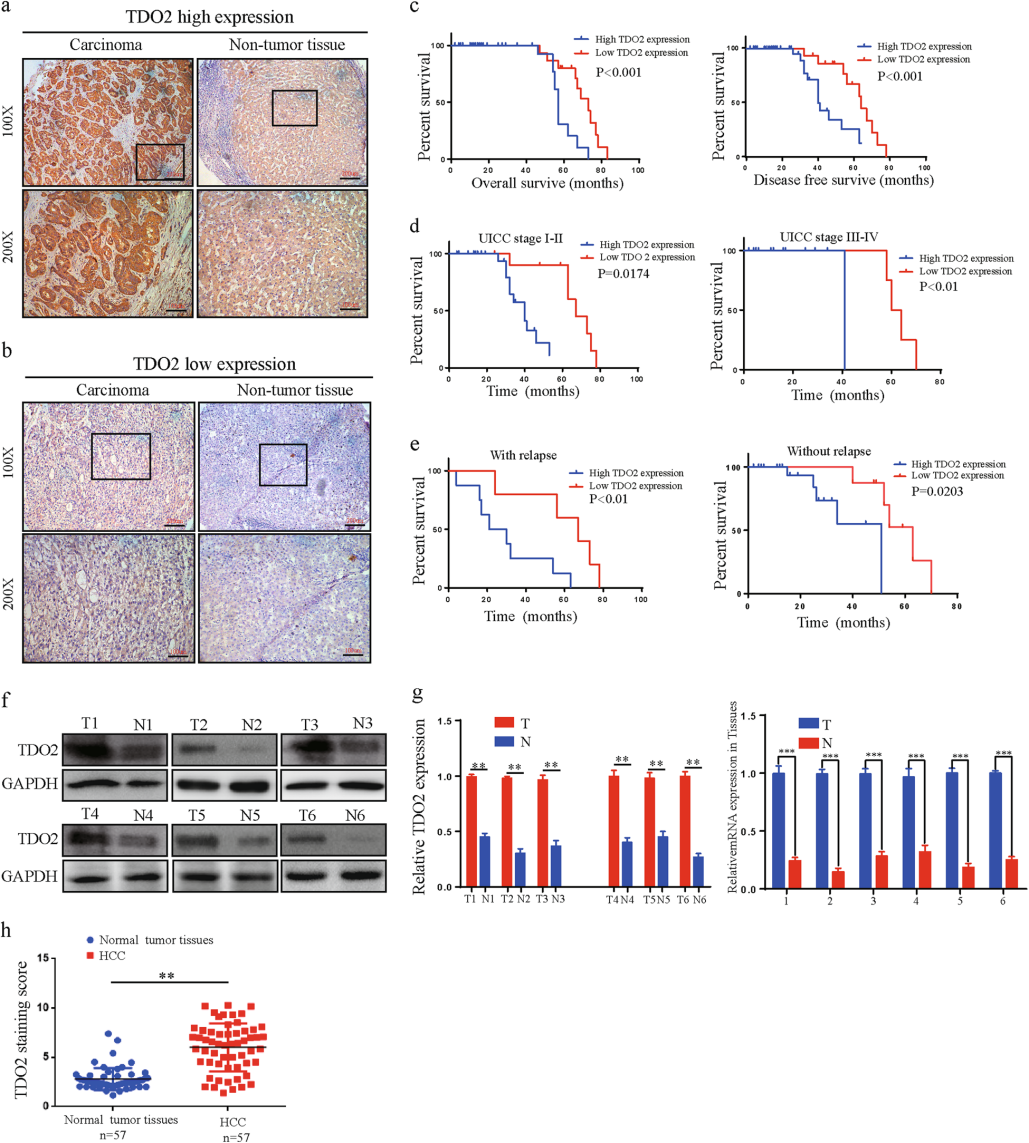
TDO2 is upregulated in HCC tissues and is correlated with progression and poor prognosis. **a**, **b** High TDO2 expression in HCC tissues as indicated by IHC. Low TDO2 expression in normal adjacent tissues as indicated by IHC. **c–e** Kaplan–Meier analysis with log-rank testing of survival was performed in HCC patients with different TDO2 expression levels (**c**), differentiation in early UICC stage (I–II) and advanced UICC stage (III–IV) (**d**), and with or without relapse (**e**). **f**, **g** Western blot analysis and qRT-PCR were used to detect TDO2 protein expression in six representative paired HCC tissue specimens. **h** Staining scores of TDO2 expression in 57 pairs of fresh frozen HCC and matched nontumor tissues. All data are from three independent experiments and are presented as the means ± SEM or representative of three independent experiments with similar results (*p < 0.05, **p < 0.01, ***p < 0.001).

Immunohistochemistry (IHC) was performed on a tissue microarray comprising 57 pairs of HCC and matched normal adjacent tissues. TDO2 expression was mainly localized to the cytoplasm and it was higher in HCC tissues than in the corresponding normal tissues (Fig. 5a, b). Thus, there was no significant difference in the numbers of normal tissue samples with low versus high TDO2 expression (*n* = 28 vs 29 respectively), whereas, the number of tumor tissue samples with low TDO2 expression (*n* = 13) was considerably lower than that (*n* = 44) of samples with high TDO2 expression (*P* = 0.006). Moreover, TDO2 overexpression was highly correlated with clinicopathological characteristics. We found that TDO2 overexpression was associated with tumor size (*p* = 0.002), tumor differentiation (*p* = 0.012), and M stage (*p* = 0.049), but there was no evidence that high TDO2 expression was associated with sex, age, tumor number, liver cirrhosis, or hepatitis virus (*p* > 0.05). Patients with high TDO2 expression had poorer OS (*p* < 0.001) and DFS (*p* < 0.001) than those with low TDO2 expression (Fig. 5c). Additionally, we observed a similar trend in which patients at UICC stage I–II (*p* = 0.0174), at UICC stage III–IV (*p* < 0.01), with relapse (*p* < 0.01) or without relapse (*p* = 0.0203) (Fig. 5d, e); with high TDO2 expression had shorter OS than the corresponding patients with low TDO2 expression, respectively. In summary, TDO2 was overexpressed in HCC tissues, which could predict poor clinical outcomes in these patients. We further explored TDO2 expression in HCC tissues and adjacent normal tissues (Table 3). The results showed that TDO2 protein and mRNA expression were upregulated in HCC tissues compared with the levels in corresponding normal tissues (Fig. 5f–h).

**Table 3:** Correlation between clinicopathologic features and the expression of TDO in 57 cases of HCC tissues (*n* = 57, *χ*^2^-test).

	N	TDO2	Expression	*P*-value
		High (%)	Low (%)	
Age (year)				0.258
<60	45	32 (71.1%)	13 (28.9%)	
≥60	12	11 (91.7%)	1 (8.3%)	
Gender				0.261
Male	46	33 (71.7%)	13 (90.9%)	
Female	11	10 (28.3%)	1 (9.1%)	
Tumor number				0.664
Single	49	36 (73.5%)	13 (26.5%)	
Multiple	8	7 (87.5%)	1 (12.5%)	
Tumor size				0.002
<5 cm	27	15 (55.6%)	12 (44.4%)	
≥5 cm	30	28 (93.3%)	2 (6.7%)	
Tumor differentiation				0.012
I–II	37	24 (64.9%)	13 (35.1%)	
III–IV	20	19 (95.0%)	1 (5.0%)	
Tumor location				0.054
Left liver	10	5 (50.0%)	5 (50.0%)	
Right liver	42	34 (81.0%)	8 (19.0%)	
Whole liver	5	4 (100.0%)	1 (0.0%)	
Vascular invasion				0.083
Yes	16	15 (93.8%)	1 (6.3%)	
No	41	28 (68.3%)	13 (31.7%)	
Liver cirrhosis				0.358
Yes	29	20 (69.0%)	9 (31.0%)	
No	28	23 (82.1%)	5 (17.9%)	
Hepatitis virus				0.629
Yes	6	4 (66.7%)	2 (33.3%)	
No	51	39 (76.5%)	12 (23.5%)	
M stage				0.049
M0	46	32 (69.6%)	14 (32.4%)	
M1	11	11 (100%)	0 (0%)	

### TDO2 promotes the progression of HCC cells

**Fig. 6:**
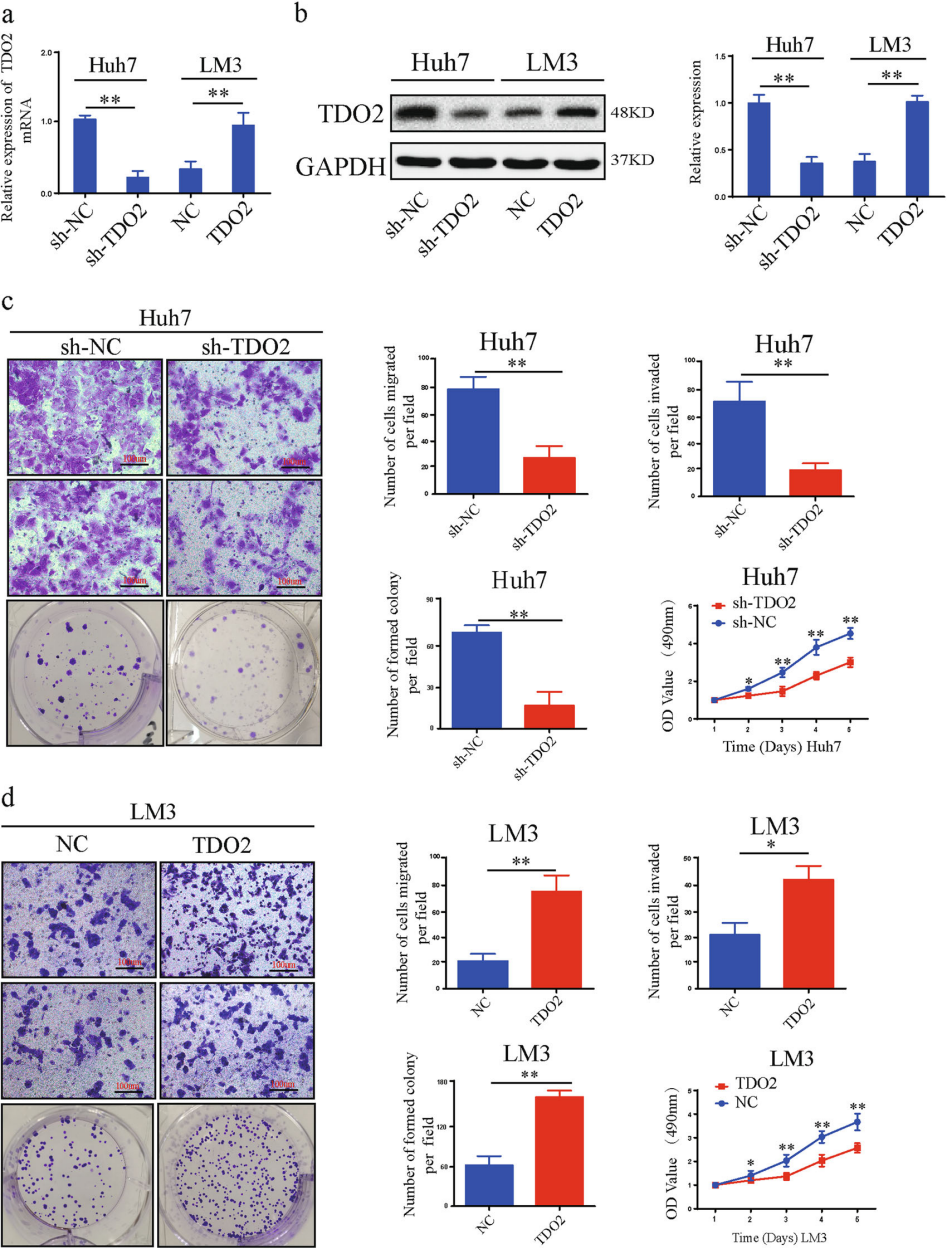
TDO2 promotes the progression of HCC cell lines. **a**, **b** Relative expression of TDO2 in Huh7 and LM3 cells transfected with shTDO2, TDO2, or NC was detected by qRT-PCR and WB. **c**, **d** The effects of TDO2 on HCC cells migration, invasion, and proliferation were evaluated by the transwell migration and invasion assays, the colony formation assay and the CCK-8 assay. All data are from three independent experiments and are presented as the means ± SEM or representative of three independent experiments with similar results (*p < 0.05, **p < 0.01, ***p < 0.001).

To detect the function of TDO2 in HCC cells, we knocked down and overexpressed TDO2 in HCC cells and then evaluated TDO expression by qRT-PCR and WB (Fig. 6a, b). Then, the functional assays were conducted and the results indicated that TDO2 promoted the mobility, migration, invasion, and proliferation of HCC cells (Fig. 6c, d Supplementary Fig. S5a, b).

### CircZNF566 promotes HCC progression via TDO2

**Fig. 7:**
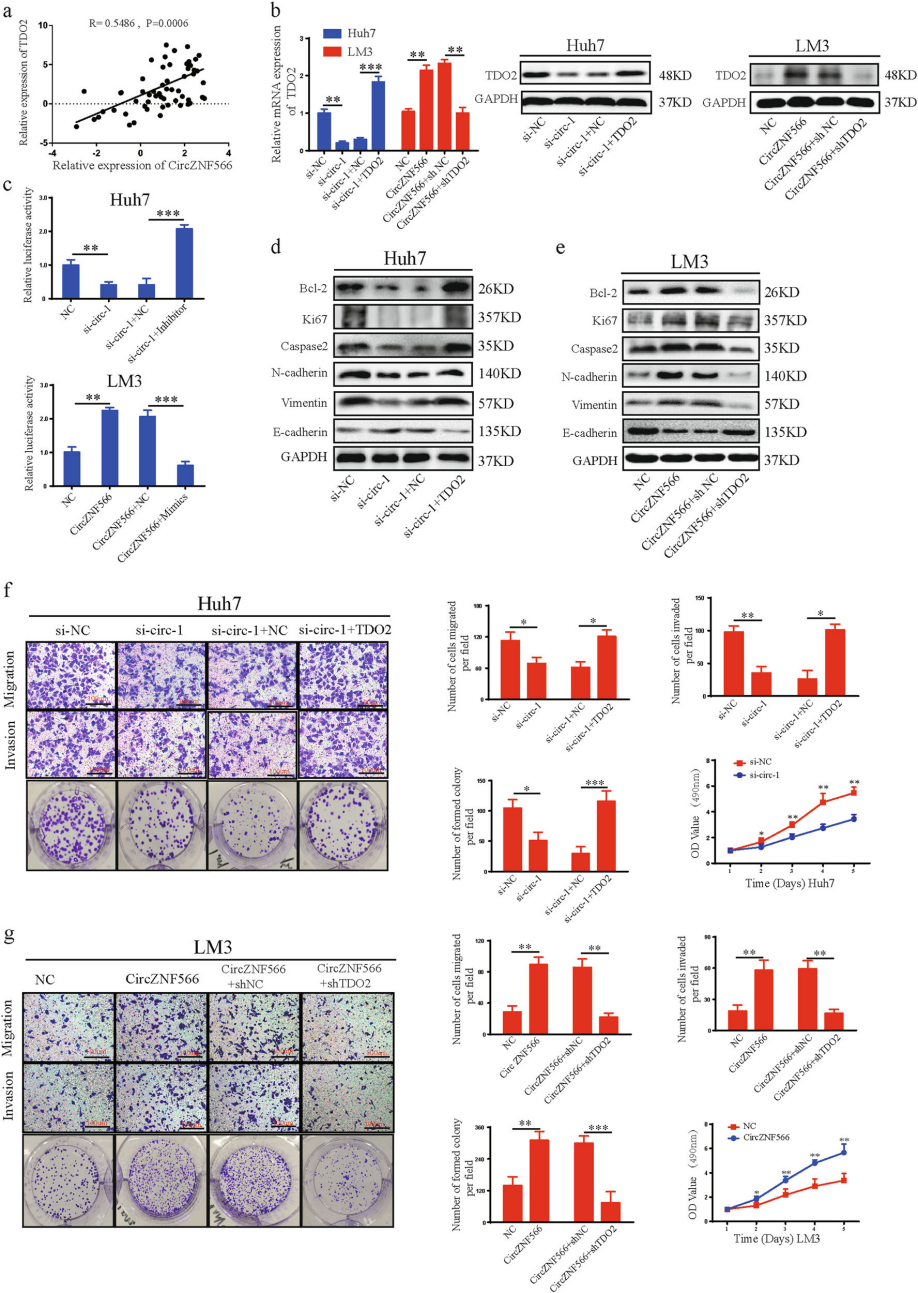
CircZNF566 promotes HCC progression via TDO2. **a** Pearson correlation analysis indicated a significantly positive correlation between the levels of circZNF566 and TDO2 in 57 pairs of HCC tissues and matched normal liver tissues. **b** Relative TDO2 expression was detected in HCC cells transfected with shTDO2, TDO2 overexpression plasmid or NC and si-circZNF566, circZNF566, or NC. **b** Relative luciferase activities were detected in HCC cells transfected luciferase reporter plasmids expressing the WT TDO2 mRNA 3′-UTR. **d**, **e** The effects of circZNF566 and TDO2 on the protein expression of Bcl-2, N-cadherin, E-cadherin, Ki67, Caspase, and Vimentin were detected by WB. **f**, **g** The effects of circZNF566 and TDO2 on HCC cells migration, invasion, and proliferation in HCC cell lines were evaluated by transwell migration and invasion, colony formation and CCK-8 assays. All data are from three independent experiments and are presented as the means ± SEM or representative of three independent experiments with similar results (*p < 0.05, **p < 0.01, ***p < 0.001).

CircRNAs may promote the expression of target genes by sponging miRNAs, according to the hypothesis of competing endogenous RNAs (ceRNAs). We found that circZNF566 functions as an efficient miR-4738-3p sponge in HCC and that TDO2 is a direct target of miR-4738-3p. Therefore, we speculated that circZNF566 promotes HCC progression via TDO2. Pearson correlation analysis indicated that circZNF566 levels were positively correlated with TDO2 in HCC tissues (Fig. 7a). Silencing circZNF566 significantly decreased TDO2 expression in both HCC cells, while TDO2 expression was increased after upregulation of circZNF566 in both cells (Fig. 7b, Supplementary Fig. S6a). Therefore, we assumed that circZNF566 positively regulated TDO2 expression. To confirm this hypothesis, a luciferase reporter plasmid with wild-type TDO2 mRNA 3’-UTR was constructed, and found that silencing circZNF566 significantly decreased the luciferase activity, while circZNF566 overexpression obviously increased the luciferase activity (Fig. 7c). In addition, miR-4738-3p reversed the ability of circZNF566 to enhance the luciferase activity of wild-type TDO2 mRNA 3’-UTR (Fig. 7c). Downregulation or upregulation of circZNF566 respectively decreased and increased the protein and mRNA expression of Bcl-2, Ki67, N-cadherin, Caspase, and Vimentin, and the protein and mRNA expression of E-cadherin was opposite in HCC cells (Fig. 7d, e, Supplementary Fig. 6b, c). These results indicated that circZNF566 positively regulates TDO2 expression.

To further explore the effects of TDO2 on the roles of circZNF566 in HCC progression, rescued experiments were conducted. TDO2 overexpression relieved the suppression of silencing circZNF566 on the expression of Bcl-2, Ki67, N-cadherin, Caspase, and Vimentin, and the E-cadherin was opposite; in addition, TDO2 knockdown reversed the effects of circZNF566 overexpression on the protein expression (Fig. 7d, e, Supplementary Fig. 6b, c). Functionally, the functional assays showed that TDO2 overexpression blocked the effects of silencing circZNF566 on the suppression of HCC cell mobility, migration, invasion, and proliferation, while downregulation of TDO2 mitigated the circZNF566-induced increases these abilities (Fig. 7f, g, Supplementary Fig. S6d, 6e). In summary, circZNF566 promotes the progression of HCC via TDO2.

### CircZNF566 promotes the growth and metastasis of xenograft tumors of HCC cells in vivo

**Fig. 8:**
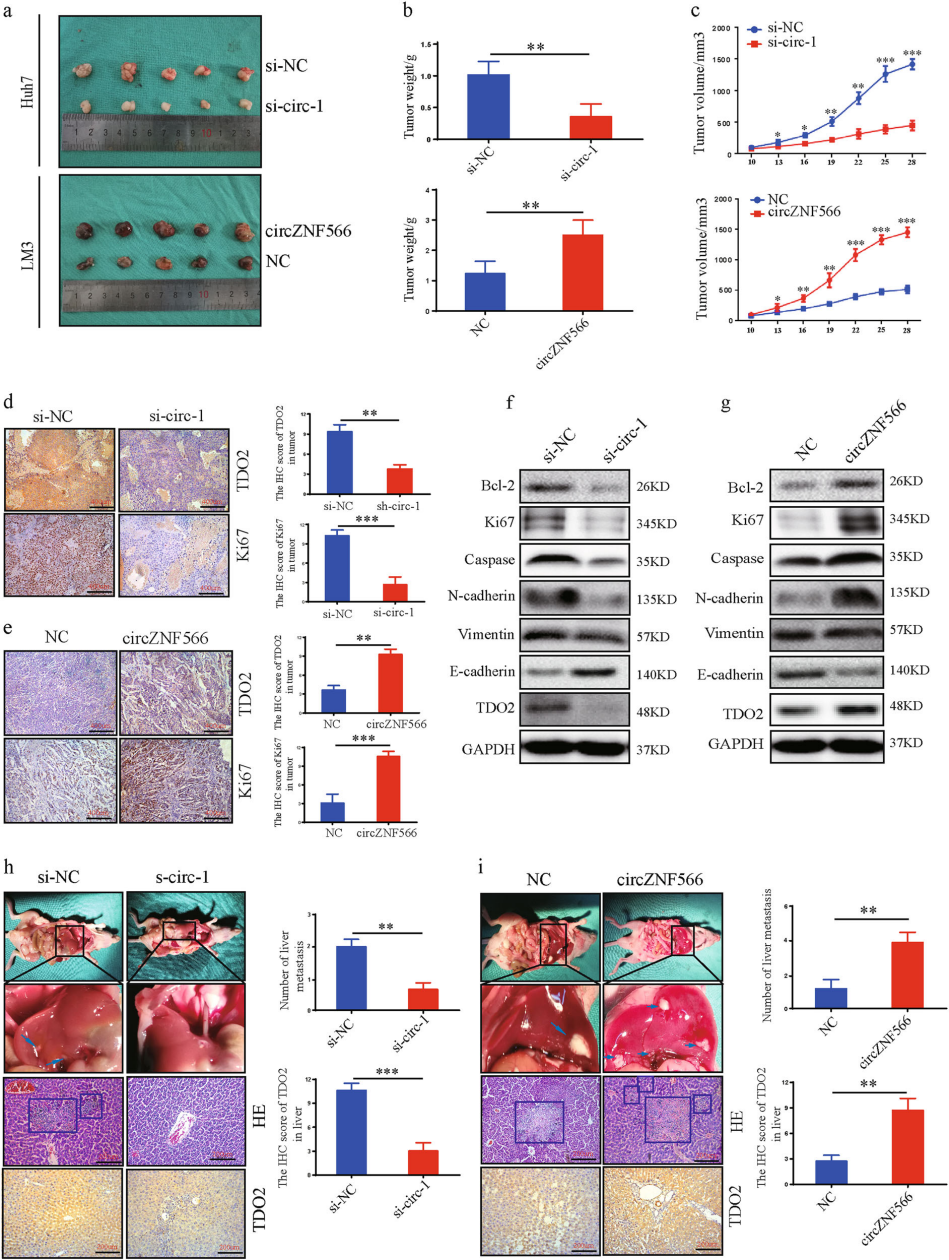
Circ ZNF566 promotes the growth and metastasis of HCC cells in vivo. **a** Images of subcutaneous xenograft tumors derived from Huh7 and LM3 cells. **b**, **c** Tumor weights of Huh7 and LM3 cells are shown, and the tumor volumes of Huh7 and LM3 cells were measured seven times every 3 days. **d**, **e** TDO2 and Ki67 expression in subcutaneous xenograft tumors of Huh7 and LM3 cells was detected by IHC. **f**, **g** The protein expression levels of TDO2, Bcl-2, Ki67, Caspase, N-cadherin, E-cadherin, and Vimentin were detected by WB in subcutaneous xenograft tumors of Huh7 and LM3 cells. **h**, **i** Images of liver metastasis assays indicating that overexpression or knockdown of circZNF566 could increase or decrease the number of liver metastasis nodules compared with the NC, respectively. H&E staining and IHC revealed liver tumor metastasis. All data are from three independent experiments and are presented as the means ± SEM or representative of three independent experiments with similar results (*p < 0.05, **p < 0.01, ***p < 0.001).

To investigate the functions of circZNF566 in vivo, a xenograft nude mouse model was established by subcutaneously injecting mice with HCC cells (*n* = 5 for each group). The stable Huh7 cells with sh-circZNF566 or sh-NC and stable LM3 cells with overexpression of circZNF566 or NC were constructed. After 28 days, all the mice were sacrificed, and the tumor samples were harvested. The weight and volume of the tumors derived from cells with circZNF566 knockdown were markedly lower than control group (Huh7 cells), while the overexpressing circZNF566 group were significantly higher than control group (LM3 cells) (Fig. 8a, b). We also found that knockdown of circZNF566 decreased the TDO2 and Ki67 expression by IHC, while overexpression of circZNF566 increased their expression in tumors (Fig. 8d, e). Profound evidence showing changes in the protein and mRNA expression levels of TDO2, Bcl-2, Ki67, Caspase, N-cadherin, Vimentin, and E-cadherin (Fig. 8f, g, Supplementary Fig. 7a, b).

To investigate the role of circZNF566 on tumor metastasis, the liver metastasis model was established. Necropsy and HE staining showed that the si-circ-1 group had significantly fewer nodules of liver metastases than the si-circ-NC group, and the NC group had significantly fewer nodules of liver metastases than the circZNF566 group (Fig. 8h, i). The TDO2 expression in the nodules of liver metastasis results showed that in the si-circ-1 group exhibited low TDO2 expression, while in the circZNF566 group exhibited high TDO2 expression, compared with the NC group (Fig. 8h, i). In conclusion, these data demonstrate that circZNF566 promotes cell growth and metastasis of HCC in vivo.

## Discussion

CircRNAs have been proven to be a type of highly stable endogenous noncoding RNA in recent years. The role of circRNAs in tumorigenesis and development has been confirmed and attracted widespread attention for the diagnosis and treatment, including Alzheimer’s disease, diabetes, lung cancer, bladder cancer, gastric cancer, and HCC^10,24–27^. However, their expression and function of circRNAs in HCC development and progression is still elusive. In this study, we identified a novel circRNA (circZNF566) and detected its expression in HCC and paired adjacent normal liver tissues, and we found that circZNF566 is significantly upregulated in HCC and is closely related to HCC progression. Because of the limited number of HCC tissue samples initially screened, there may be some other important upregulated circRNAs that also participate in the development of HCC and need to be investigated in the future^28–30^.

We assessed the expression profile of circRNAs in HCC tissues and found that circZNF566 was the most obviously upregulated circRNA and that it was related to metastasis. For further verification, we found that circZNF566 expression was increased significantly in HCC and was correlated with UICC T stage, lymphatic metastasis, distant metastasis, and poor prognosis, indicating that circZNF566 might function as a tumor promoter and be associated with the carcinogenesis and progression of HCC. In addition, circZNF566 promotes the mobility, migration, invasion, and proliferation of HCC cells in vivo and in vitro. Moreover, circZNF566 has a stable loop structure, which corresponds to strong resistance to exonuclease activity and actinomycin D. CircRNAs are an essential type of ceRNAs, and could act as miRNA sponges to affect gene regulation and expression. CircRNAs usually attenuate the inhibitory effects of miRNAs on their target genes, and an increasing number of studies have confirmed this phenomenon of regulating posttranscriptional function^31,32^. In our study, we found that miR-4738-3p had a high binding capacity with circZNF566 in HCC cells. TDO2 contains the MRE of miR-4738-3p, which plays a key role in HCC progression and metastasis. RIP and luciferase assays have been shown to efficiently identify the precise and authentic interactions between circZNF566 and miR-4738-3p^9,33^. We found that miR-4738-3p directly suppresses TDO2 expression by binding to the 3′-UTR of TDO2 mRNA. Therefore, we are the first to report the role of the circZNF566/miR-4738-3p/TDO2 axis in HCC progression.

The level and function of miR-4738-3p in HCC are unclear. We first demonstrated that miR-4738-3p was downregulated in HCC tissues, was negatively correlated with clinicopathological features and poor prognosis and inhibited the progression of HCC, all of which suggest that miR-4738-3p acts as a tumor suppressor. We further analyzed miR-1306-3p activity and found that it could reverse the effects of circZNF566 on promoting TDO2 expression and the mobility, migration, invasion, and proliferation of HCC, while TDO2 blocked the ability of miR-4738-3p to suppress these activities. This result indicated that circZNF566 could promote HCC progression by serving as a miR-4738-3p sponge and by inhibiting its suppressive activities on the expression of the target gene TDO2.

TDO2 is a homotetrameric cytosolic enzyme^14,34,35^ that is expressed mainly in only the liver; it is the rate-limiting enzyme in the first step of Try metabolism in mammals and converts Try to produce Kyn^36,37^. The use by tumors of proinflammatory Kyn metabolites such as 3-hydroxykynurenine, 3-hydroxyanthranilic acid and quinolinic acid to undermine the immune system and achieve immune escape^38–40^. TDO2 was first found to be expressed in human glioma cells and to mediate the Try-Kyn-Ahr pathway, impacting tumor and immune biology regulation; this regulation of the immune response in tumors is exhibited by many human tumors^21,22,41,42^. TDO2 overexpression was associated with cancer stem cells and poor prognosis in esophageal squamous cell carcinoma and TDO2 was significantly upregulated in metastatic leiomyosarcoma tumors compared with the levels in primary tumors. Nevertheless, the detailed functions and mechanisms of TDO2 in HCC cells remain unknown^21,43,44^. In our study, we are the first to identify TDO2 as a driver gene in HCC through loss-of-function experiments. We determined that TDO2 is upregulated in HCC tissues and promotes the mobility, migration, invasion, and proliferation of HCC cells. Therefore, by enhancing the transcriptional activity of TDO2 and by sponging miR-4738-3p, circZNF566 promotes both the progression and metastasis of HCC via the circZNF566/miR-4738-3p/TDO2 axis.

Some circRNAs are stably expressed in serum and in other cells or tissues, which makes them suitable biomarkers for certain diseases as well as targets for diagnosis, prognosis, and treatment. Other studies have indicated that circRNAs play a crucial role in the progression and prognosis of human cancer. Therefore, circZNF566 may also play an important role and need further investigation to verify that it is present in serum or in other tissues. Moreover, many unknown circRNAs and their functions in the development and progression of cancers remain to be discovered. Accordingly, more endeavors and further studies are needed to elucidate the functions and mechanisms of circRNAs in cancers.

## Conclusion

In summary, our findings reveal that circZNF566 expression is significantly upregulated in HCC and is correlated with poor prognosis in HCC patients. Functionally and mechanistically, circZNF566 promotes HCC progression and metastasis via the circZNF566/miR-4738-3p/TDO2 axis, indicating its tumor promoter role in HCC development. Furthermore, the in vivo intervention of circZNF566 indicates its potential in HCC-targeted therapies. Our data suggest that circZNF566 possesses considerable potential as a prognostic predictor and therapeutic target for HCC.

## Materials and methods

### Patient tissue samples, cell lines, and culture

The 57 pairs of HCC and paired adjacent liver tissues were collected from patients during operation at Shanghai General Hospital (Shanghai, China) who hadn’t received chemotherapy, radiotherapy or other related anti-tumor therapies prior to the surgery. The experiments were approved by the Institutional Research Ethics Committee of Shanghai General hospital of Shanghai Jiao Tong University and the ethical guidelines of Helsinki^45^. These tissues were collected following surgical resection and stored at −80°C immediately for further RNA and protein extraction, and immediately fixed in formalin to construct tissue microarray (TMA). The normal liver cell line Lo2 and seven hepatoma cell lines (Huh7 and QSG-7701, Hep3B, HepG2, MHCC-97L, MHCC97H, and MHCC-LM3) were obtained from the Cell Bank of Shanghai Institutes of Biological Sciences, Chinese Academy of Sciences (Shanghai, China). Cell lines were authenticated using Short Tandem Repeat (STR) analysis and tested for mycoplasma contamination. All cell lines were cultured in Dulbecco’s modified Eagle’s medium (Invitrogen, Grand Island, NY) containing 10% fetal bovine serum and 1% penicillin streptomycin (Gibco, USA) in 5% CO_2_ at 37 °C.

### RNA extraction, Nuclear-cytoplasmic fractionation, RNase R, Actinomycin D treatment, and quantitative real-time PCR (qRT-PCR)

RNA extraction and qRT-PCR were performed as previously described^9^. Nuclear and cytoplasmic RNA fractionation was isolated with PARIS™ Kit (Invitrogen, USA) following the manufacturer’s instruction. For RNase R treatment, 10 μg total RNA was incubated for 15 min at 37 °C with 40 U RNase R (Epicentre Technologies, Madison, USA). For circRNA and mRNA, RNA was reverse transcribed into cDNA using a PrimeScriptTM RT Master Mix reagent kit (TaKaRa, Shiga, Japan). For miRNA, cDNA was synthesized by the PrimeScriptTM RT reagent kit (TaKaRa, Shiga, Japan). Transcription was prevented by the addition of 2 mg/ml Actinomycin D or DMSO (Sigma–Aldrich, St. Louis, MO, USA) as the negative control. After treatment with Actinomycin D and RNase R, the RNA expression levels of ZNF566 and circZNF566 were detected by qRT-PCR; cDNA was used as a template for q RT-PCR with SYBR Premix Ex Taq II (TaKaRa, Shiga, Japan). Normalization with Glyceraldehyde-3-phosphate dehydrogenase (GAPDH) or U6 were used as internal controls for circRNA, mRNA or miRNA. The copy number of each PCR product was three, and the relative levels were calculated by the 2-ΔΔCt method. Three experiments were performed with three replicates each.

### Western blotting analysis

The HCC tissues or cells were lysed by RIPA buffer and the protease inhibitor phenylmethanesulphonyl fluoride was added for 30 min at 4 °C (Beyotime Biotechnology, China). BCA protein assay kit (Beyotime Biotechnology, China) was used to detect the protein concentration. SDS-PAGE sample loading buffer (Beyotime Biotechnology, China) was used to separate the protein. Protein was transferred onto PVDF membranes (Millipore, Billerica, MA) after electrophoresis. PVDF membranes were blocked in 5% milk-TBST at room temperature, then washed by TBST and incubated with primary antibodies at 4 °C overnight. The primary antibodies included TDO2 antibody (1:500, Novus, USA), Ki67 (1:200, Cell Signaling Technology, USA), Vimentin (1:2000, Abcam, USA), Bcl-2(1:2000, Abcam, USA), E-cadherin (1:2000, Abcam, USA), Caspase (1:1000, Cell Signaling Technology, USA), N-cadherin(1:2000, Abcam, USA), PCBP2 (1: 1000, MBL, Japan), GAPDH monoclonal antibody (1:2000, Proteintech, USA), and β‐actin (1: 10000, Sigma‐Aldrich, USA). After the membranes were washed by TBST, secondary antibody was added and incubated for 2 h at room temperature. The secondary antibodies included goat anti-rabbit IgG (1:2000, Proteintech, USA) and goat anti-mouse IgG (1:2000, Proteintech, USA). After washing three times, proteins were detected by ECL regent (Millipore, Billerica, MA).

### Transfection, oligonucleotides and plasmids

To regulate circZNF566, miR-4738-3p, and TDO2 expression, oligonucleotides and plasmids were constructed. The following siRNAs targeting circZNF566 were designed by RiboBio (Guangzhou, China): si-circ-1 target, 5’-AGAGAATTCACACAGTCCT-3’; si-circ-2 target, 5’- TCACACAGTCCTGGAATCA -3’; and si-circ-3 target, 5’-CACAGTCCTGGAATCAAGA-3’. Full-length circZNF566 was cloned into the pEX-3 (GenePharma, Shanghai, China) overexpression vector. The mimics, inhibitor and negative controls for hsa-miR-4738-3p were purchased from RiboBio (Guangzhou, China). The shRNA-TDO2 sequences were as follows: 5’-CGAGCACTGACCAGGATTA-3’ (target), 5’- CGCACCCAGGAGCGAGCT-3’ (sense), and 5’-GCGACCTCTTCTGAGCTGA-3’ (antisense). The TDO2 gene was cloned into pLVX plasmids (HarO Life, Shanghai, China). The oligonucleotides and plasmids were transfected into cells with Lipofectamine 2000 (Invitrogen, Carlsbad, CA, USA) according to the manufacturer’s instructions.

### Luciferase reporter assay

The luciferase reporter plasmids (pLuc-Firefly-Renilla containing circZNF566 sequence and Mutant sequence, pLuc-Firefly-Renilla containing TDO2 sequence and Mutant sequence) were synthesized by Gene Chem Co (Shanghai, China). Huh7 and LM3 cell lines were transfected with the luciferase reporter plasmids by Lipofectamin2000 (Invitrogen, Carlsbad, CA, USA) and incubated for 24 h. Then, firefly luciferase activity was normalized to Renilla luciferase activity. The effects of a miR-4738-3p on the luciferase reporter with the circ ZNF566 3′-UTR, TDO2 3′-UTR or the corresponding mutant was calculated by comparing the reporter with the control. All experiments were independently repeated in triplicate.

### Pull-down assay with a biotinylated circZNF566 probe

Pull-down assay was performed as indicated^33^. Firstly, 1 × 107 cells (Huh7 and LM3) were collected, lysed, and sonicated. Probe-coated beads were generated by co-incubating the circZNF566 probe with C-1 magnetic beads (Life Technologies) at room temperature for 2 h. Secondly, the cell lysates were incubated with the circZNF566 probe or oligo probe at 4 °C overnight. Lastly, after washing with wash buffer, the RNA complexes bound to the beads were eluted and extracted with the RNeasy Mini Kit (QIAGEN) for RT-PCR or qRT-PCR. The biotinylated circZNF566 probe was designed and synthesized by RiboBio (Guangzhou, China).

### RNA immunoprecipitation (RIP) assay

RIP assay was performed using the Magna RIP RNA-Binding Protein Immunoprecipitation Kit (Millipore, MA, USA) as described. Hun7 cells were transfected with the miR-4738-3p mimics or negative control. The cells were lysed in complete RNA lysis buffer after 48 h. The negative control was normal mouse IgG (Beyotime, China), and the positive control was human anti-AGO2 antibody (Millipore, Billerica, USA). The antibodies were added into cell lysates and rotated overnight. After incubating with Proteinase K buffer for 30 min the next day, the immunoprecipitated RNAs were isolated and extracted by Trizol reagent (Invitrogen, USA). Then the RNAs were detected by qRT-PCR and agarose gel electrophoresis performed to identify the expression of circZNF566 and miR-4738-3p.

### Haematoxylin and eosin (HE) and immunohistochemistry

Under sterile conditions, the tissues immersed in 10% neutral formalin and fixed for 24 h. Subsequently, the tissues were dehydrated, embedded in paraffin and serially sectioned to 4-μm sections. HE staining was performed using standard procedures. Immunohistochemistry was performed as described. Before antigen retrieval in citrate buffer, TMAs was dewaxed and rehydrated and a graded series of ethanol. Then, it was incubated with TDO2 antibody (1:500, Novus, USA), Ki67 (1:200, Cell Signaling Technology, USA) overnight at 4 °C, followed by exposing with an HRP-conjugated secondary antibody for 30 min at 25 °C. Staining intensity for TDO and Ki67 were score as: 0 (negative), 1 (weak), 2 (moderate), and 3 (intense). Staining area was scored as: 0 (0), 1 (1–25%), 2 (26–50%), 3 (51–75%), and 4 (76–100%). After multiplying the staining intensity score by the staining area score, the total score index was designated as follows: 0–3, negative expression; 4–6, weak expression; and 8–12, strong expression^46,47^. The intensity and extent of staining was scored independently by two experienced pathologists who did not know information of patients.

### Cell proliferation

HCC cells were seeded in 96-well plates (2 × 103 cells per well), each well containing 100 ml medium. After culturing cells for 0, 1, 2, 3, and 5 days, 10 ul Cell Counting Kit-8 (CCK8) (Dojindo, Japan) was added to each well at six time points, cells were then incubated for additional 2 h. Finally, the absorbance was measured at 450 nm by Varioskan LUX (Thermo Fisher, CA, USA). The experiments were performed as previously described and run in three times.

### Plate colony formation assays

Colony formation assay Transfected cells were seeded in 6-well plate and then cultured for 14 days. The cells were washed with phosphate-buffered saline (PBS) and then fixed with 4% paraformaldehyde for 15 min and stained with crystal violet solution for 20 min. Then, colonies cells were count and the plates were photographed. The experiments were performed as previously described and run in three times.

### Wound healing assays

HCC cells were cultured in 6-well plates (1.0 × 105 cells/ well). After 12 h, a uniform scratch was made down the center of the well with a 200 μl pipette tip, and then, the cells were rinsed once with phosphate-buffered saline. Cells were cultured in serum-free medium during the experimental periods. Representative images of cell migration were captured by photographing 10 high-power fields at 0 and 24 h after injury, and wound widths were quantified and compared to baseline values. The experiments were performed as previously described and run in three times.

### Transwell migration and invasion assays

The transwell 24-well Boyden chamber (Corning, USA) with 8.0 μm pore size polycarbonate membrane was used for the cell migration (without) Matrigel (BD Bioscience, USA) and invasion assays (with) Matrigel assays according to the manufacturer’s protocol. Briefly, each group of cells (5 × 104/chamber) was plated in the upper chambers in 200 ul serum-free media for 24 h, while the bottom chambers contained 600 ul media supplemented with 10% fetal bovine serum (FBS) as a chemoattractant. Cells that migrated and invaded to the reverse side of chamber inserts were fixed by 4% polymethanol for 30 min and stained with 0.1% crystal violet for 30 min. The experiments were performed as previously described and run in three times

### Tumor formation assay in nude mice

Four-week-old male BALB/C nude mice were randomly divided into four groups (*n* = 5) and maintained under specific pathogen-free conditions with a 12-h light/dark cycle. Huh7 or LM3 cells were subcutaneously injected into the right flank of the nude mice with 1.0 × 107 cells from stable cells lines. Tumor size was monitored twice a week, and tumor volume was estimated using the following formula: volume = width^2^ × length × π/6. All the mice were sacrificed 4 weeks and xenografts were removed and weighed, then fixed in 4% paraformaldehyde. Tumor weight is shown as the mean ± SEM of each group. For liver metastasis assay, cells were injected into the spleen of nude mice (4-week-old mice; with 1 × 107 cells per mouse), after 8 weeks post injection, livers were photographed and foci numbers on the surface were counted followed by standard H&E procedure^48^. Liver foci were counted by microscope. All animal experiments were undertaken in accord with the National Institutes of Health Guide for the Care and Use of Laboratory Animals, with the approval of the Institutional Animal Care and Use Committee of Shanghai General Hospital.

### Statistical analysis

Statistical analyses were performed with SPSS 20 software (SPSS, Chicago, IL, USA). Data were analyzed with unpaired Student’s *t*-test unless indicated otherwise. The correlations were analyzed by Pearson’s test (r, P). The *χ*^2^ test was appropriately used to determine the statistical significance between TDO expression and clinicopathological variables. Survival curves were calculated by the Kaplan–Meier method with the Log-rank test employed for the comparison of differences. The results are presented as the mean ± SEM. For all tests, *P*-values <0.05 were considered statistically significant.

## Supplementary information

Figure S1

Figure S2

Figure S3

Figure S4

Figure S5

Figure S6

Figure S7

Supplementary Figure Legends

Supplementary PCR Seguence

## Data Availability

The data that support the findings of this study are available from the corresponding author upon reasonable request.
