# Supplementary figures and images for: Circular RNA circZNF566 promotes hepatocellular carcinoma progression by sponging miR-4738-3p and regulating TDO2 expression
Source: Cell Death Dis. 2020 Jun 12;11(6):452. doi: 10.1038/s41419-020-2616-8 (PMC7293356; doi:10.1038/s41419-020-2616-8)

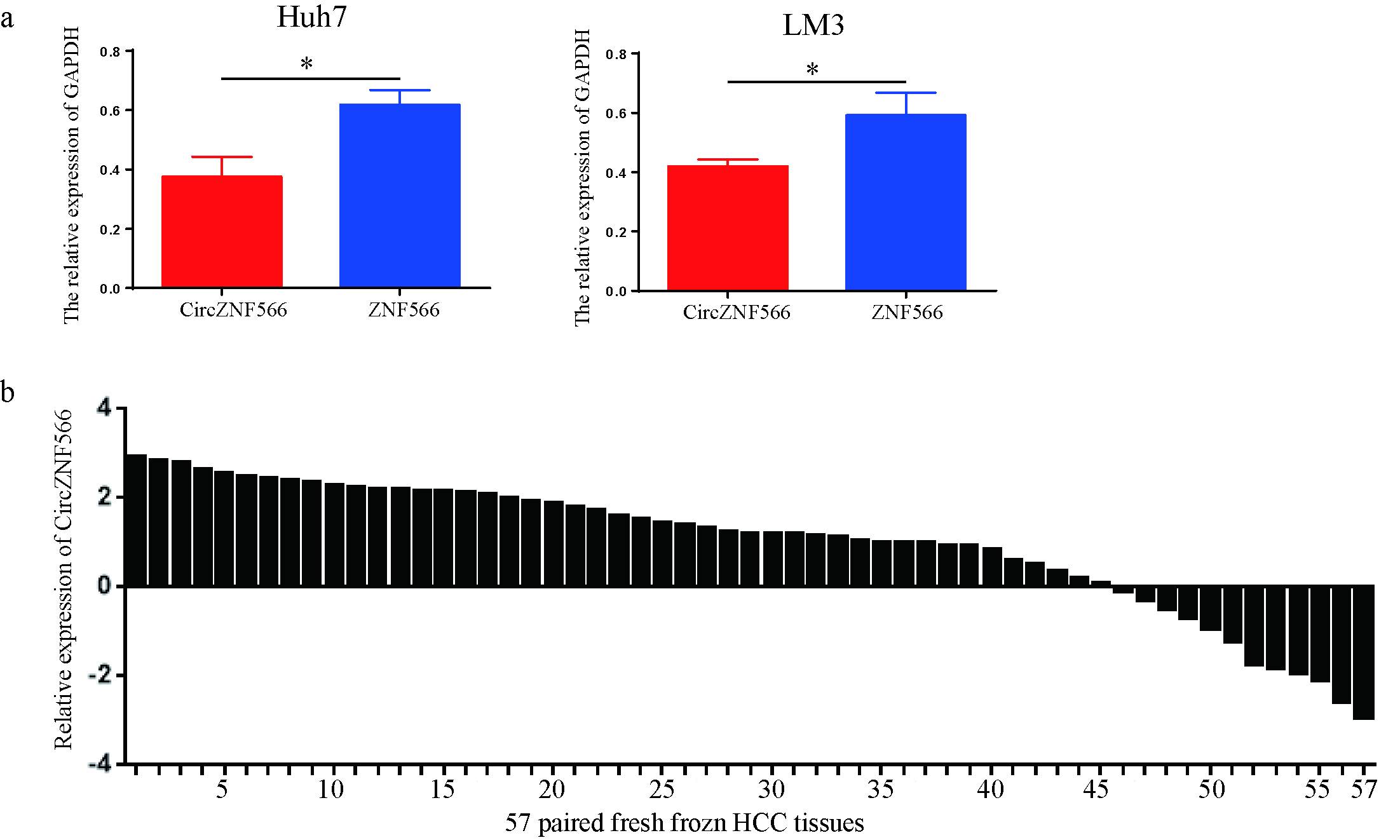

Supplement: Supplementary file 1 — Figure S1 [file 41419_2020_2616_MOESM1_ESM.tif]

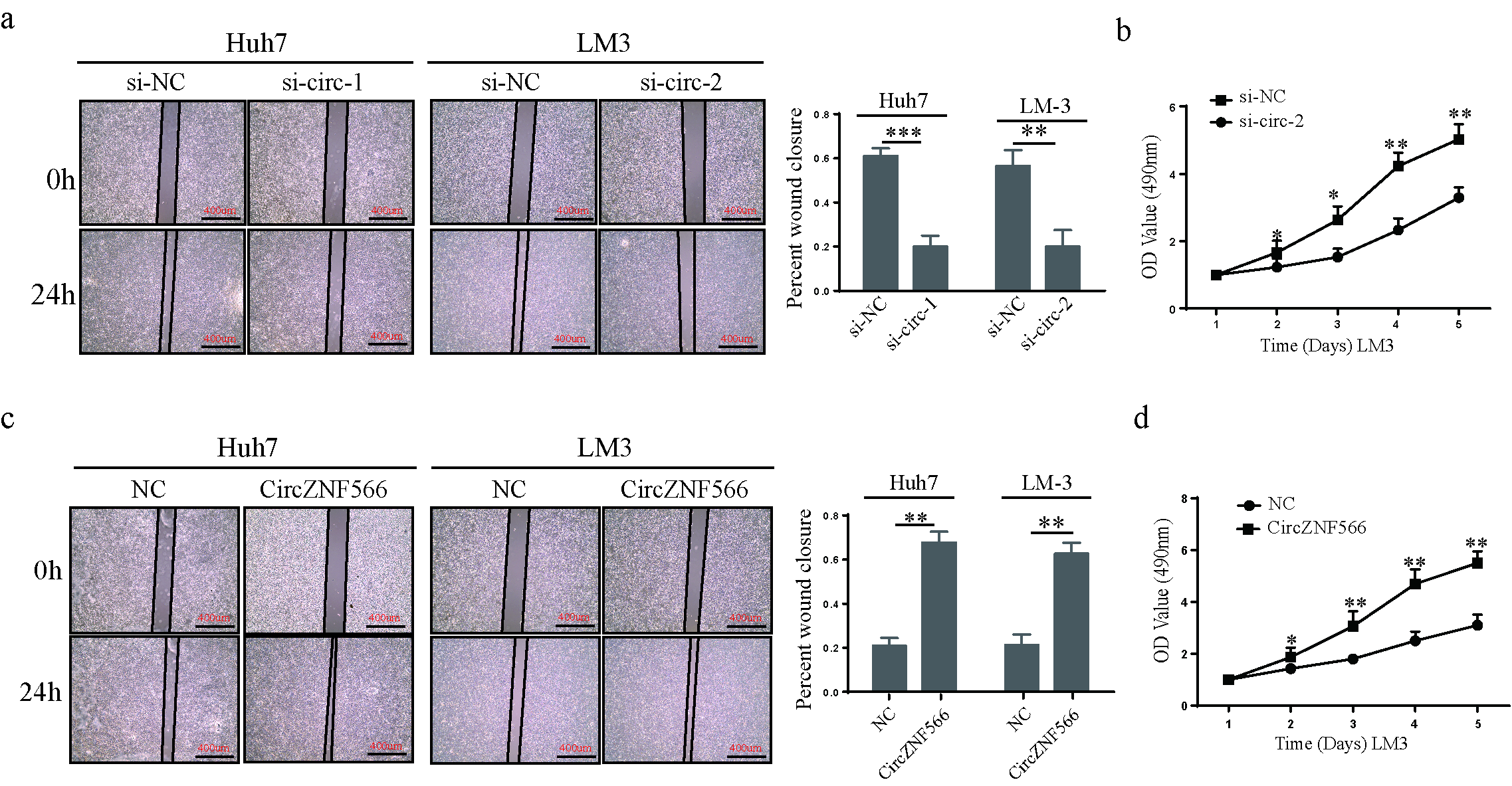

Supplement: Supplementary file 2 — Figure S2 [file 41419_2020_2616_MOESM2_ESM.tif]

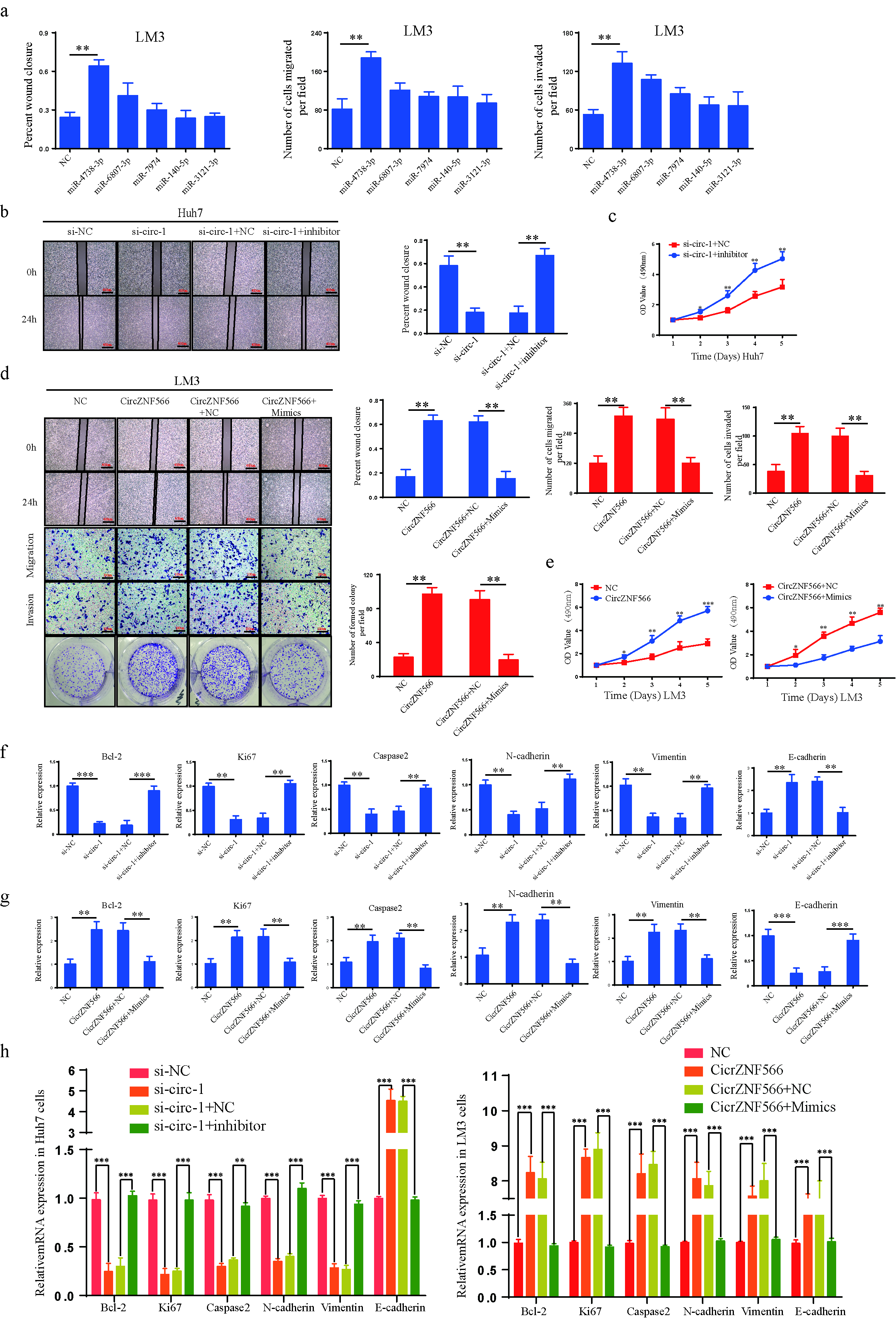

Supplement: Supplementary file 3 — Figure S3 [file 41419_2020_2616_MOESM3_ESM.tif]

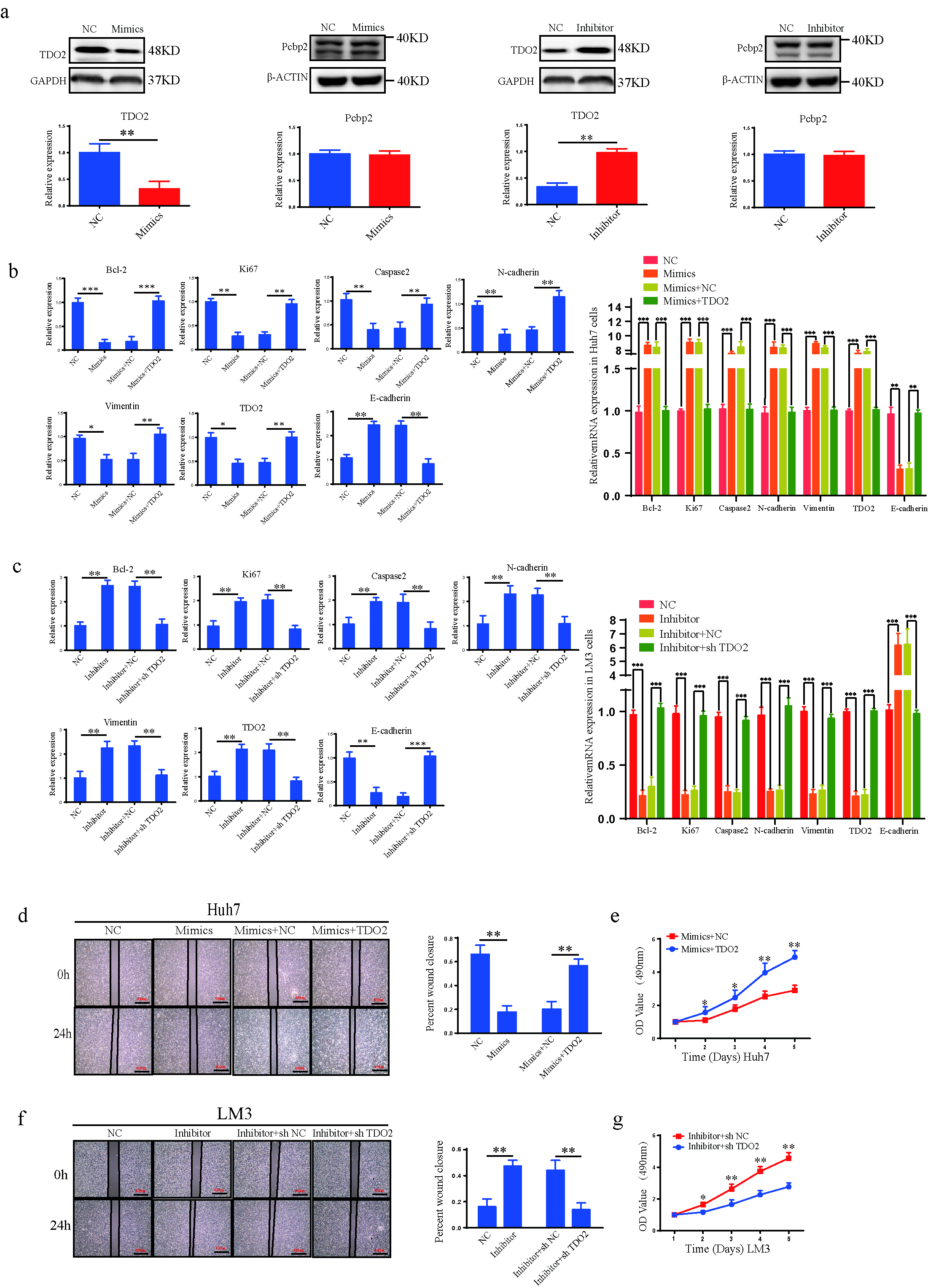

Supplement: Supplementary file 4 — Figure S4 [file 41419_2020_2616_MOESM4_ESM.tif]

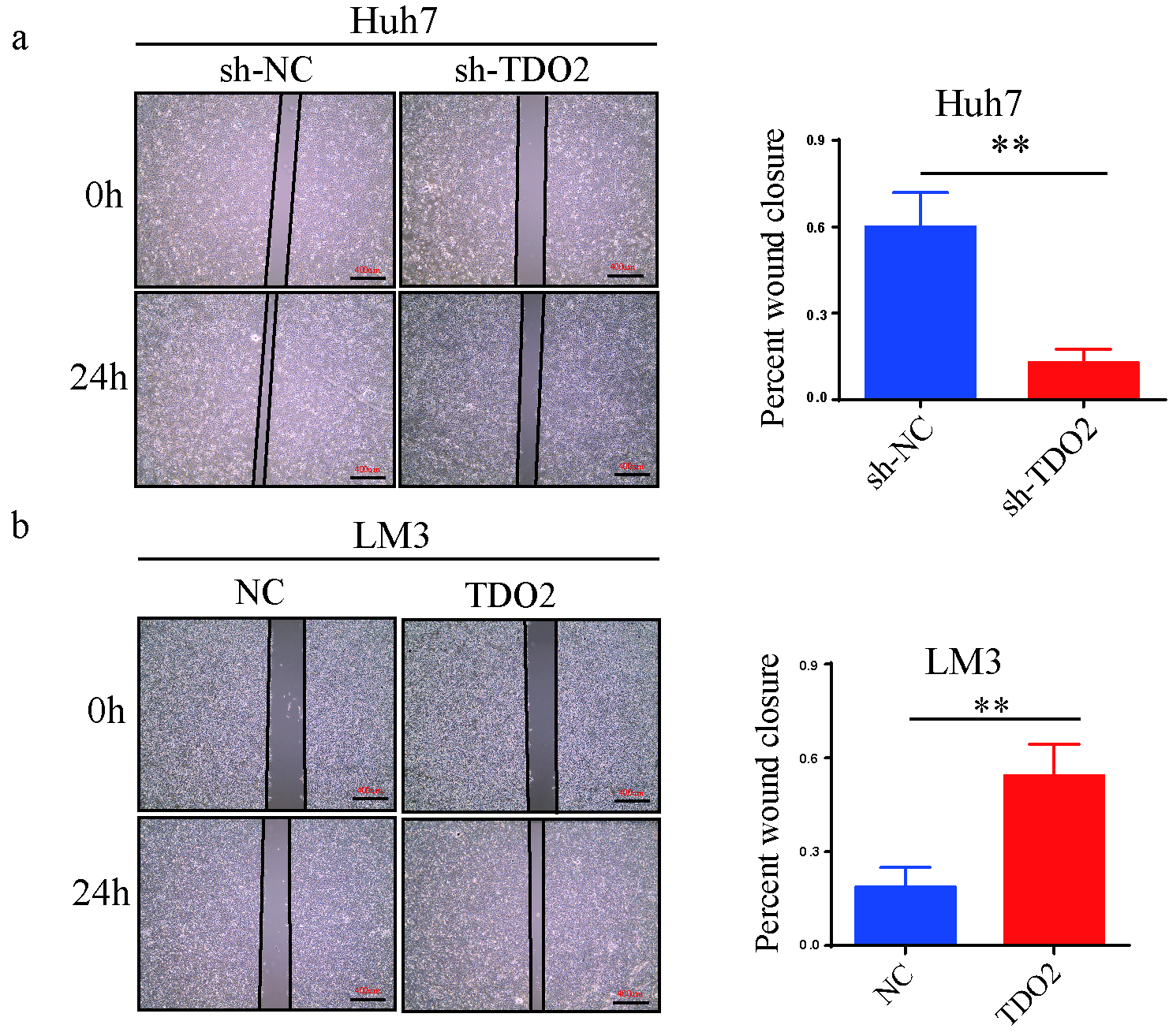

Supplement: Supplementary file 5 — Figure S5 [file 41419_2020_2616_MOESM5_ESM.tif]

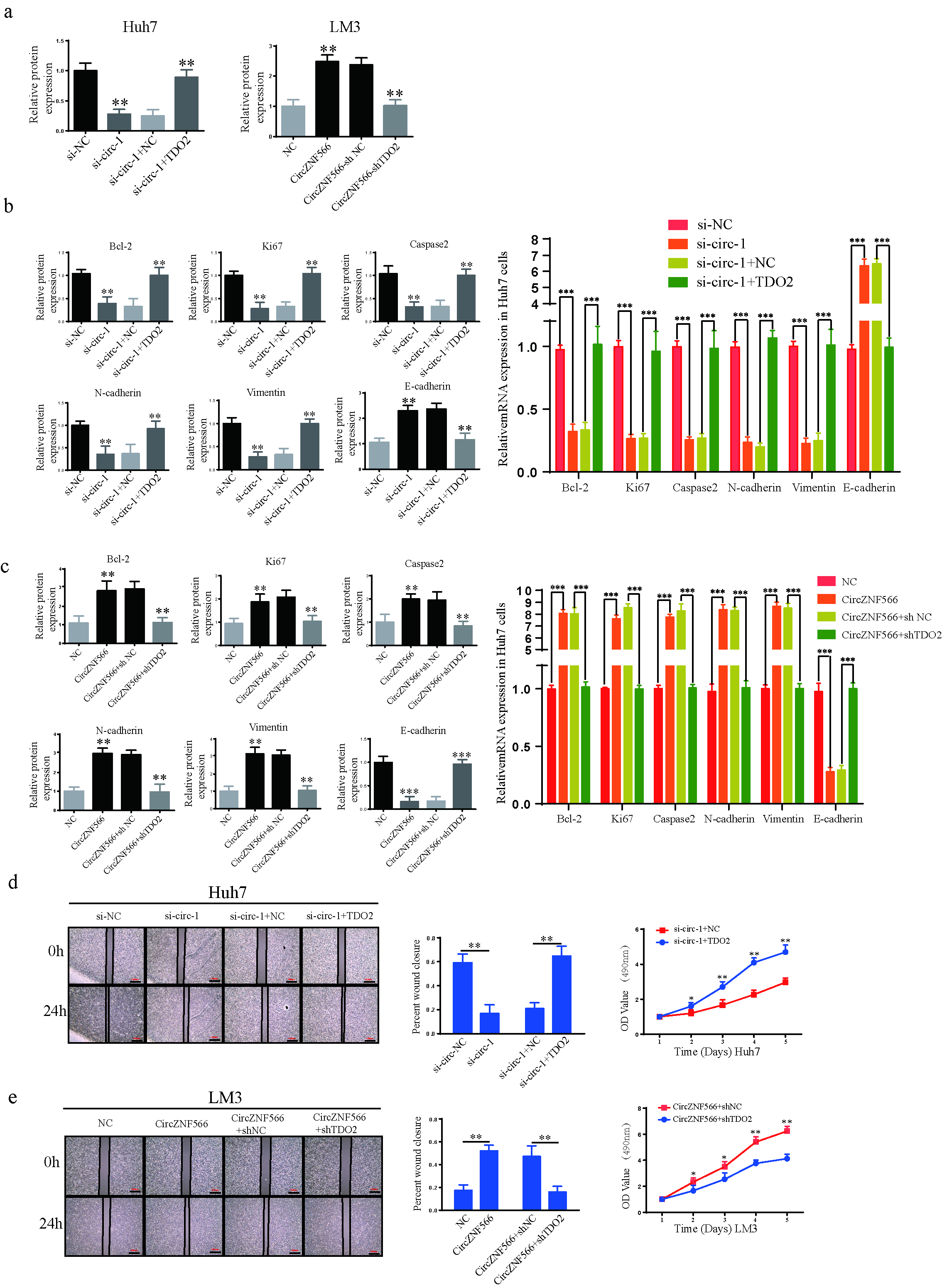

Supplement: Supplementary file 6 — Figure S6 [file 41419_2020_2616_MOESM6_ESM.tif]

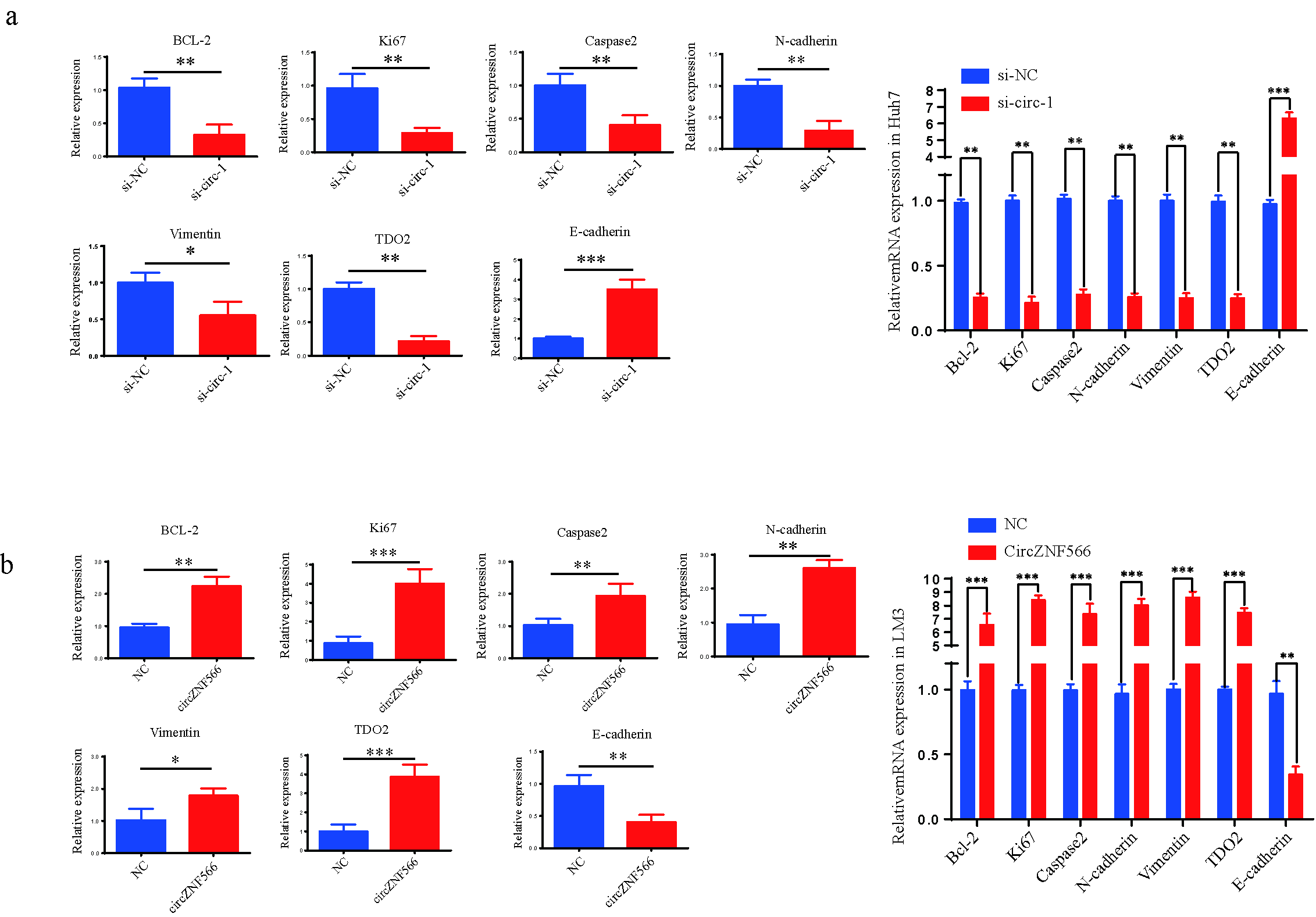

Supplement: Supplementary file 7 — Figure S7 [file 41419_2020_2616_MOESM7_ESM.tif]
