# Supplementary material for: Circular RNA circZNF566 promotes hepatocellular carcinoma progression by sponging miR-4738-3p and regulating TDO2 expression
Source: Cell Death Dis. 2020 Jun 12;11(6):452. doi: 10.1038/s41419-020-2616-8 (PMC7293356; doi:10.1038/s41419-020-2616-8)
Supplement: Supplementary file 8 — Supplementary Figure Legends [file 41419_2020_2616_MOESM8_ESM.docx]

**Figure s1** The relative quantification of ZNF566 mRNA and circZNF566 and relative circZNF566 expression in HCC.

**a** The relative quantification of the two products of the same gene (ZNF566 mRNA and circZNF566) by qRT-PCR in HCC cells (p < 0.05). **b** Relative circZNF566 expression in 57 pairs of fresh frozen HCC tissues and matched normal liver tissues by qRT-PCR. All data are from three independent experiments and are presented as the means ± SEM. (*p < 0.05)

**Figure s2** CircZN566 promotes the mobility, and proliferation of HCC cells.

**a** Cell mobility in Huh7 and LM3 cells with knockdown of circZNF566 were evaluated by the wound healing assay. **b** Proliferation in LM3 cells with knockdown of circZNF566 were evaluated by the CCK-8 assay. **c** Cell mobility in Huh7 and LM3 cells that overexpressed circZNF566 were evaluated by the wound healing assay. **d** Proliferation in LM3 cells that overexpressed circZNF566 were evaluated by the CCK-8 assay. All data are from three independent experiments and are presented as the means ± SEM. (*p < 0.05, **p < 0.01, ***p < 0.001.)

**Figure s3** CircZNF566 promotes HCC progression by serving as a miRNA sponge of miR-4738-3p.

**a** Wound healing and transwell migration and invasion assays were performed to analyze the cell mobility, migration and invasion of LM3 cells transfected with 5 selected miRNA mimics and NC mimics. **b** and **c** The effects of circZNF566 and miR-4738-3p on Huh7 cells mobility proliferation were evaluated by wound healing and CCK-8 assays. **d** and **e** The effects of circZNF566 and miR-4738-3p on LM3 cells mobility, migration, invasion and proliferation were evaluated by wound healing, transwell migration and invasion, colony formation and CCK-8 assays. **f**, **g** and **h** The effects of circZNF566 and miR-4738-3p on the protein and mRNA expression levels of Bcl-2, Ki67, Caspase2, N-cadherin, E-cadherin, and Vimentin in Huh7 and LM3 cells were detected by WB and qRT-PCR. All data are from three independent experiments and are either presented as the means ± SEM or representative of three independent experiments with similar results. (*p < 0.05, **p < 0.01, ***p < 0.001.)

**Figure s4** MiR-4738-3p suppresses HCC progression by directly targeting TDO2.

**a** TDO2 and Pcbp2 expression were detected by WB and qRT-PCR in HCC cells treated with mimics, inhibitor or NC. **b** and **c** The effects of miR-4738-3p and TDO2 on the protein and mRNA levels of TDO2, Bcl-2, Ki67, Caspase2, N-cadherin, E-cadherin, and Vimentin were detected by WB and qRT-PCR. **d** and **e** The effects of miR-4738-3p and TDO2 on Huh7 cells mobility and proliferation were evaluated by wound healing and CCK-8 assays. **f** and **g** The effects of miR-4738-3p and TDO2 on LM3 cells mobility and proliferation were evaluated by wound healing and CCK-8 assays. All data are from three independent experiments and are presented as the means ± SEM or representative of three independent experiments with similar results. (*p < 0.05, **p < 0.01, ***p < 0.001.)

**Figure s5** TDO2 promotes the progression of HCC cell mobility.

**a** and **b** The effects of TDO2 on cells mobility and proliferation were evaluated by the wound healing assay. All data are from three independent experiments and are presented as the means ± SEM or representative of three independent experiments with similar results. (**p < 0.01)

**Figure s6** CircZNF566 promotes HCC progression via TDO2.

**a** Relative TDO2 expression was detected in HCC cells transfected with shTDO2, TDO2 overexpression plasmid or NC and si-circZNF566, circZNF566 or NC. **b** and **c** The effects of circZNF566 and TDO2 on the protein and mRNA levels of Bcl-2, Ki67, Caspase2, N-cadherin, E-cadherin, and Vimentin were detected by WB and qRT-PCR. **d** and **e** The effects of circZNF566 and TDO2 on cell mobility and proliferation in HCC cell lines were evaluated by wound healing and CCK-8 assays. All data are from three independent experiments and are presented as the means ± SEM or representative of three independent experiments with similar results. (*p < 0.05, **p < 0.01, ***p < 0.001.)

**Figure s7**. Protein expression levels in subcutaneous xenograft tumors derived from Huh7 and LM3 cells.

**a** and **b** The protein and mRNA levels of TDO2, Bcl-2, Ki67, Caspase2, N-cadherin, E-cadherin, and Vimentin were detected by WB and qRT-PCR. All data are from three independent experiments and are presented as the means ± SEM or representative of three independent experiments with similar results. (*p < 0.05, **p < 0.01, ***p < 0.001.)
