# Supplementary material for: Circular RNA circZNF566 promotes hepatocellular carcinoma progression by sponging miR-4738-3p and regulating TDO2 expression
Source: Cell Death Dis. 2020 Jun 12;11(6):452. doi: 10.1038/s41419-020-2616-8 (PMC7293356; doi:10.1038/s41419-020-2616-8)
Supplement: Supplementary file 9 — Supplementary PCR Seguence [file 41419_2020_2616_MOESM9_ESM.docx]

Table S1. The sequences of primers for qRT-PCR.

| CircZNF566 |  | Sense  Antisense | GACATCAGAGAATTCACACAGTCCT  GAAACTGGAGCACTGAAAATCAC |
| --- | --- | --- | --- |
| TDO2 |  | Sense  Antisense | CTCCGTGCTTCTCAGACAGTGC  TTGTCTTCTTCGCTGCCTTCTACG |
| ZNF566 |  | Sense  Antisense | TTCTCTCAGGAGGAGTGGGAATGC  AGAATGCCCTGCCATTGAAACCAG |
| Pcbp2 |  | Sense  Antisense | AGGCAGGTTACCATCACTGG  CATTGTTCTAGCTGCTCCCC |
| Bcl-2 |  | Sense  Antisense | GTCATGTGTGTGGAGAGCGTCAACC  CCAGGGCCAAACTGAGCAGAGTC |
| Ki67 |  | Sense  Antisense | AGGACTTTGTGCTCTGTAACC  CTCTTTTGGCTTCCATTTCTTC |
| Caspase-2 |  | Sense  Antisense | GGTGATGGTCCTCCCTGTCT  TACTCATCACCAGTGCCAAGC |
| E-cadherin |  | Sense  Antisense | GGCCTGAAGTGACTCGTAACGA  CAGCCGCTTTCAGATTTTCATC |
| N-cadherin |  | Sense  Antisense | TCAGTGGCGGAGATCCTACT  TGGTTTGACCACGGTGACTA |
| Vimentin |  | Sense  Antisense | CGAAACTTCTCAGCATCACG  GCAGAAAGGCACTTGAAAGC |
| GAPDH |  | Sense  Antisense | GGGAAGGTGAAGGTCGGAGT GGGGTCATTGATGGCAACA |
| miR-4378-3p |  | stem-loop  Sense  Antisense | GTCGTATCCAGTGCAGGGTCCGAGGTATTCGCACTGGATACGACTCCTCC  CGAGAAACAGGAGCGCCA  AGTGCAGGGTCCGAGGTATT |
| U6 |  | stem-loop  Sense  Antisense | CTCAACTGGTGTCGTGGAGTCGGCAATTCAGTTGAGAAAAATAT  CAAGGATGACACGCAAA  TCAACTGGTGTCGTGG |
